# COSMIN review of the PANSS Marder factor solution and other factor models in people with schizophrenia

**DOI:** 10.1038/s41537-025-00600-6

**Published:** 2025-03-31

**Authors:** Maximilian Roithmeier, Simon Geck, Markus Bühner, Sophia Wehr, Lucia Weigel, Josef Priller, John M. Davis, Stefan Leucht

**Affiliations:** 1 Department of Psychiatry and Psychotherapy, School of Medicine and Health, Technical University of Munich, Klinikum rechts der Isar, Ismaningerstrasse 22, 81675 Munich, Germany; 2 Department of Psychology, Ludwig Maximilian University of Munich, Leopoldstr. 13, 80802 Munich, Germany; 3 German Center for Mental Health (DZPG) site Munich Augsburg, Munich, Germany; 4 Neuropsychiatry and Laboratory of Molecular Psychiatry, Charité – Universitätsmedizin Berlin and DZNE, Berlin, Germany; 5 University of Edinburgh and UK DRI, Edinburgh, UK; 6 Psychiatric Institute, University of Illinois at Chicago (mc 912), 1601 W. Taylor St., Chicago, IL 60612 USA

**Keywords:** Schizophrenia, Psychosis

## Abstract

The Positive and Negative Syndrome Scale (PANSS) is widely used to assess schizophrenia symptoms. Initially designed with three subscales, Marder et al.´s 5-factor-Model (M5M) first proposed in 1997 has been frequently used in treatment trials, but it has never been systematically reviewed for its measurement properties. We utilized the COnsensus-based Standards for the selection of health Measurement INstruments (COSMIN) guideline for systematic reviews and meta-analytical procedures to assess the psychometric properties of the M5M-PANSS. COSMIN comprises several steps: literature search, risk-of-bias assessments, assessing the updated criteria for good measurement properties, feasibility aspects and grading the quality of the evidence. We further assessed the goodness of fit of other PANSS factor models. We included 95 publications. The M5M-PANSS showed good construct validity, but “insufficient” structural validity. Evidence of other COSMIN domains is largely lacking. Among the multiple (73) factor solutions examined with confirmatory methods, several other 5-factor solutions had better model fit. According to COSMIN rules the M5M should not be recommended for use. Other five-factor models such as the one proposed by Wallwork et al.^1^ warrant further evaluation. Nevertheless, the factor composition of the M5M and these other models was relatively similar, so previously published results should not be disregarded.

## Introduction

The Positive and Negative Syndrome Scale (PANSS)^2^ is the standard instrument for assessing symptoms of schizophrenia. Initially, the PANSS was developed by Kay et al.^2^ as a three-subscale instrument consisting of a positive subscale with 7 items, a negative subscale with 7 items and a third general psychopathology subscale with 16 items. However, Kay and Sevy^3^ later hypothesized a pyramidical structure within the scale and Peralta and Cuesta^4^ proposed a five-factor structure in 1994. Another five-factor model was published by Marder et al.^5^ in 1997. This Marder-5-factor-Model (M5M), comprises the following factors: (1) positive factor, (2) negative factor, (3) disorganized thought factor, (4) uncontrolled hostility/excitement factor, (5) anxiety/ depression factor^5^. Multiple other factor analyses have been conducted since and most found similar, but not identical 5-factor models. The M5M is especially important as it has established itself especially in antipsychotic drug trials. Le Moigne et al.^6^ for example, quote 15 such trials. However, the measurement properties of the M5M-PANSS^5^ have never been systematically reviewed.

We have already conducted a COSMIN systematic review on the measurement properties of the PANSS in its original three-subscale structure^7^.

However, it must be considered that, even though the same items are used, according to e-mail correspondence with COSMIN^8–10^, the scale needs to be treated as a separate instrument. Findings of the original subscales cannot be simply transferred to the M5M factors, because certain measurement properties of a scale are dependent on its structure.

Our first objective is to comprehensively review and provide insight into the measurement properties of the M5M-PANSS^5^ by applying the COnsensus-based Standards for the selection of health Measurement INstruments (COSMIN) guideline for systematic reviews of patient-reported outcome measures^8–10^ including meta-analytic methods when appropriate.

Our second objective is to assess the goodness of fit of further PANSS factor models and compare it with the M5M.

## Methods

**Fig. 1:**
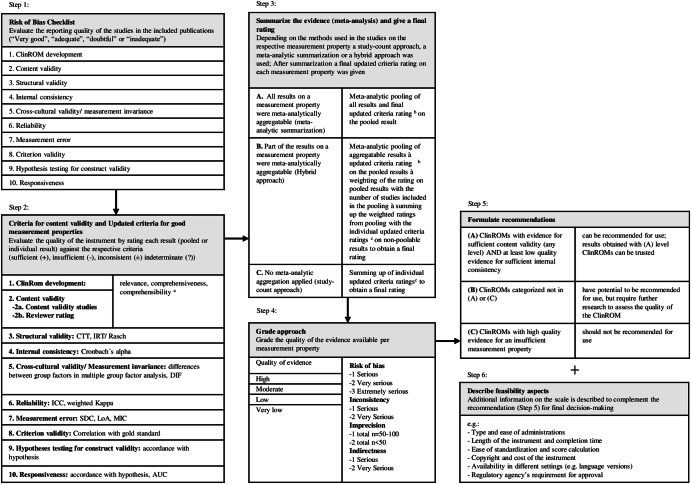
Overview of the COSMIN methodology including adaptions for our research situation. Refer to the methods part and appendix of our publication for further information. For the unmodified, full version of the COSMIN methodology, refer to the COSMIN manual. Step 1: Risk of bias checklist. Step 2:*Criteria for content validity* and *Updated criteria* for good measurement properties.^a^For the detailed criteria refer to the COSMIN manual for content validity. Step 3: Summarize the evidence and give a final rating. ^b^The same criteria like in Step 2 apply. ^c^These are the individual ratings on each result obtained in Step 2. Step 4: Grade approach. Step 5: Formulate recommendations. Step 6: Describe feasibility aspects.

The methods used in this review are based on the COSMIN guideline for systematic reviews of patient-reported outcome-measures^8–10^. COSMIN was initially created for patient-reported outcome-measures (PROMs) but can also be applied to clinician-reported outcome-measures (ClinROMs)^11,12^, such as the PANSS. There are several steps: literature search, quality assessment of the individual analyses with the COSMIN *Risk-of-Bias checklist*, applying *updated criteria for good measurement properties*, grading the overall quality of evidence with a modified Grading of Recommendations assessment, development and evaluation (GRADE) approach, assessing feasibility aspects and formulating a recommendation. An overview is presented in Fig. 1.

Data extraction sheets as recommended by COSMIN (compare COSMIN manual, pp. 66–73) were used^8–10^.

All steps of the COSMIN methodology were conducted independently by two reviewers (SG and MR). Any disagreements were resolved by consensus, with the involvement of a third, professor-level reviewer (SL) if necessary. We followed the same procedure as in our COSMIN review of the PANSS in its original three-subscale structure^7^.

In COSMINs terminology the term “studies” is used for individual analyses^8–10^. We referred to individual analyses as “analyses” or “assessments” and publications as “studies” to improve comprehensibility and readability.

We investigated the M5M^5^ for its importance in antipsychotic drug trials, but we also examined other 5-factor solutions found in the literature and their fit.

A protocol was registered on the Open-Science-Foundation website where more details can be found (10.17605/OSF.IO/5EGMD; compare Appendix 1)^13^.

### Search strategy and selection criteria

PhD librarian Farhad Shokraneh searched PubMed and EMBASE using a modified COSMIN filter, see Appendix 2 and Terwee et al.^14^. The search term used according to COSMIN was: ‘PANSS’OR ‘Positive and Negative Syndrome Scale‘ AND ‘Schizophrenia‘ AND [COSMIN inclusion-filter] NOT [COSMIN exclusion-filter]. We screened the identified titles and abstracts, and reference lists of included publications for evaluation studies of the M5M-PANSS^5^ in which at least 80% patients had a psychotic disorder in any stage (e.g. acute or in remission). Articles that addressed the model fit of other PANSS-models (e.g. 7-factor solutions or 5-factor solutions other than M5M-PANSS) were also included.

There were no language or country restrictions. Following COSMIN, only full-text articles were included, thus conference abstracts were excluded. Reasons for the exclusion of articles as well as the process of screening the literature search results can be found in a PRISMA diagram in Appendix 3. If uncertainties arose regarding results or methods of relevant articles, their authors were contacted and asked to revalidate the results or processes. This applied to 29 articles.

### Assessing the risk of bias (step 1 in Fig. 1)

The reporting quality of the analyses included was assessed with the COSMIN risk of bias checklist which consists of 10 domains (called “boxes” by COSMIN): (1) *ClinROM development*, (2) *content validity*, (3) *structural validity*, (4) *internal consistency*, (5) *cross-cultural validity*/ *measurement invariance*, (6) *reliability*, (7) *measurement error*, (8) *criterion validity*, (9) *hypothesis testing for construct validity*, (10) *responsiveness*^8–10^. Each box consists of two to eight items rated as either “very good”, “adequate”, “doubtful”, “inadequate” or “not applicable”, a *worst score counts* principle is applied for each domain.

### Assessing the criteria for content validity and the updated criteria for good measurement properties (step 2 in Fig. 1)

The results of each analysis are evaluated following COSMIN’s *criteria for I. content validity* (Fig. 1, step 2, domains 1 and 2) or II. the *updated criteria for good measurement properties*^8–10^. (Fig. 1, step 2, domains 3–10) depending on the applicable domain. An analysis’ evidence for a respective measurement property is rated either “sufficient” (+) or “insufficient” (−), with a third option for an “indeterminate” rating (?), if neither “sufficient” nor “insufficient” ratings are applicable. Only for *content validity* (domains 1 and 2) a fourth rating option of “inconsistent” (±) is available.

The overall rating for I. *Content validity* is generally made up of three individual ratings for the *ClinROM development* study, *content validity* analyses and a third rating based on the reviewers (SG,MR,SL) own evaluation of the scales *content validity* (compare pp. 53–59 COSMIN *content validity* manual^8–10^).

The II. *Updated criteria* address domains 3–10. Concerning domain 3 *structural validity* the COSMIN guideline does not provide criteria for factor models resulting from exploratory methods^8–10^. We therefore used the same criteria we created for our COSMIN review of the Kay three-subscale PANSS^7^, adapted for the M5M^5^ for the purpose of comparing the exploratory derived models to the M5M. Our approach followed Elsman et al.^15^ who processed exploratory factor analyses (EFAs) and principal component analyses (PCAs) in the same way. The criteria are: (1) ≥50% explained variance, (2) factor loadings >0.30, (3) ≤10% cross loading items, (4) five-factor model, and (5) ≥80% of the items distributed as in the M5M^5^. A “sufficient” rating was only given if all the criteria regarding the exploratory derived models were met. Otherwise, the respective rating was “insufficient”. For the assessment of confirmatory factor analyses, the *Updated criteria*^8–10^ provide the criterion of comparative fit index (CFI)^16^ >0.95 or root mean square error of approximation (RMSEA)^17,18^ <0.06 for fit indices. We processed normed fit indices (NFI)^19^ as we did with CFIs^20^.

For assessing domains 9 *construct validity* and 10 responsiveness COSMIN requires the formulation of hypotheses. The hypotheses are: 1. Between instruments measuring the same or a very similar construct, we expected a correlation of ≥0.5, 2. Between instruments measuring related but different constructs, we expected a correlation of ≤0.6 and 3. Between instruments measuring dissimilar or contrary constructs, we expected a correlation of ≤0.4.

We considered correlations of the M5M total score and its factor-scores with other scales, correlations of single PANSS items were not considered. For details about the assessment of all other domains suggested by COSMIN see pp. 28–29 of the COSMIN manual^8–10^.

### Statistical method

In situations where few analyses are available, a simple analysis-count approach is sufficient in COSMIN reviews, see e.g. Weigel et al.^11^, Wehr et al.^12^ or Zúñiga Le-Bert et al.^21^. We summarized measures of *construct validity* with standard random-effects meta-analyses using Comprehensive Meta-Analysis (CMA) Version 2^22^. In situations where it was not possible to aggregate *all* data for a measurement property of the M5M-PANSS^5^ or its factors, we used the same hybrid approach as in our Kay PANSS COSMIN review^7^: the *updated criteria* rating of the pooled result was multiplied by the number of analyses included in the pooling to give them the appropriate weight, and summed up with ratings of analyses that could not be aggregated and were therefore rated individually.

### Grading the quality of evidence (step 4 in Fig. 1)

The COSMIN modified GRADE approach applies to the accumulated evidence on each measurement property, spanning 4 domains: (1) risk of bias, (2) inconsistency (3) imprecision and (4) indirectness^8–10^.

The final quality of the evidence is graded as high, moderate, low, or very low (Table 1a). Initially, evidence is assumed to be of high quality. If the criteria of the domains are not fulfilled, the evidence is then downgraded by one to three levels (see Table 1b).

**Table 1:** GRADE approach.

(a) Quality of evidence definitions	
High	We are very confident that the true measurement property lies close to that of the estimate of the measurement property
Moderate	We are moderately confident in the measurement property estimate: the true measurement property is likely to be close to the estimate of the measurement property, but there is a possibility that it is substantially different
Low	Our confidence in the measurement property estimate is limited: the true measurement property may be substantially different from the estimate of the measurement property
Very low	We have very little confidence in the measurement property estimate: the true measurement property is likely to be substantially different from the estimate of the measurement property
(b) Instructions on downgrading	
*Risk of bias*	
No	There are multiple analyses of at least adequate quality, or there is one study of very good quality available
−1 Serious	There are multiple analyses of doubtful quality available, or there is only one study of adequate quality
−2 Very serious	There are multiple analyses of inadequate quality, or there is only one study of doubtful quality available
−3 Extremely serious	There is only one study of inadequate quality available
*Inconsistency*	
No	Results could be meta-analytically pooled or ≥75% of the analyses had consistent results
−1 Serious	<75% of analyses had consistent results
−2 Very serious	<70 and ≥65% had consistent results If <65% had consistent results GRADE will not be performed because there is not enough evidence
*Imprecision*ª	As recommended by COSMIN due to the high total sample sizes no downgrading for imprecision was done
*Indirectness*	As recommended by COSMIN due to our inclusion criteria of ≥80% population with a psychotic disorder no downgrading for indirectness was done

ªDowngrading for Imprecision is not applicable for measurement properties in which the sample size is already evaluated in the “Risk of bias” step (analyses on content validity, structural validity and cross-cultural validity/measurement invariance).

### Recommendations (step 5 in Fig. 1)

According to COSMIN^8–10^, an overall judgment of (A) (can be recommended for use and results obtained with the scale can be trusted), (B) (potential for recommendation but needs further research into the measurement properties) or (C) (should not be recommended for use) was made.

Recommendations are mainly dependent on the evidence for *content validity* and *internal consistency*. (compare Fig. 1, step 5).

### Assessment of feasibility aspects (step 6 in Fig. 1)

To supplement the recommendations, we compiled information on the feasibility of the PANSS. Examples for the type of information assessed by COSMIN are presented in Fig. 1.

### Assessment of other five-factor models

We assessed *structural validity* of all other factor structures validated by confirmatory factor analyses in terms of goodness of fit. We compared the item distribution of 5-factor models which reached fit indices defined as “sufficient” by COSMIN^8–10^, i.e. CFI > 0.95 or RMSEA < 0.06, in at least one analysis. As the COSMIN criteria are very stringent in this regard, we additionally described the item distribution of 5-factor models reaching a more relaxed criterion of CFI ≥ 0.90 or RMSEA ≤ 0.08 which are frequently used in the literature^23,24^. We counted how often “sufficient” goodness of fit was reached by each model. We compared the item distribution of these models with one another and with the M5M^5^ to understand similarities and differences. As this final step was exploratory and descriptive, following Elsman^15^ we did not take cross-loadings into equation in our comparison and just assorted the items to the factors they loaded the highest on.

We restricted our assessment of other models to goodness of fit because in 23 included studies confirmatory assessments for 73 other factor models were identified (see results). Thus, a full COSMIN approach would not have been feasible. Moreover, all these examinations were conducted to 5-factor models only because models with fewer or more factors were comparably rare.

## Results

### Literature search

The literature search yielded 8070 results. Their title-abstract screening identified 508 articles as relevant for full-text screening. Through full-text screening 90 publications were included in the review. The additional screening of references resulted in 5 further inclusions. Therefore, the total of included publications was 95 (compare Appendix 3 PRISMA Diagram of the Search).

The included studies comprised populations of patients with schizophrenia-spectrum disorders in various disease states, with a median patient count per study of *N* = 270, a median mean age of 35.6 years and a median percentage of female participants of 34.1%. The median average disease duration was 11.6 years. Our review includes studies involving both inpatients and outpatients (quantitative distribution not reported by most authors). PANSS assessments were conducted in over 15 different languages. Most assessments were conducted in English (58%), French (12%) or Spanish (9%). For comprehensive characteristics of the study populations, please refer to Appendix 4.

### Risk of bias

Risk-of-bias evaluates the reporting quality of the analyses included (not the actual results) and is presented in Table 2.

**Table 2:** COSMIN risk of bias and criteria results.

Measurement property	Scale (no. of analyses assessing the scales measurement property)	COSMIN risk of bias				COSMIN updated criteria ratings*			
		Very good	Adequate	Doubtful	Inadequate	+^a^	−^b^	?^c^	±^d^
ClinROM** development	M5M total score (*n* = 1)	0	0	1	0	0	0	0	1
Content validity	M5M total score (*n* = 0)	0	0	0	0	0	0	0	0
Structural validity	M5M total score (*n* = 128)	29	54	11	34	12	90	26	
	Positive factor (*n* = 3)	0	3	0	0	0	0	3	
	Negative factor (*n* = 5)	2	3	0	0	2	0	3	
	Disorganized thought factor (*n* = 3)	0	3	0	0	0	0	3	
	Uncontrolled hostility/excitement (*n* = 3)	0	3	0	0	0	0	3	
	Anxiety/depression factor (*n* = 3)	0	3	0	0	0	0	3	
Hypothesis testing for construct validity	M5M total score (*n* = 5)	0	0	5	0	0	5	0	
	Positive factor (*n* = 17)	4	1	12	0	12	5	0	
	Negative factor (*n* = 17)	4	1	12	0	13	4	0	
	Disorganized thought factor (*n* = 12)	0	0	12	0	9	3	0	
	Uncontrolled hostility/excitement (*n* = 12)	0	0	12	0	9	3	0	
	Anxiety/depression factor (*n* = 12)	0	0	12	0	10	2	0	
Responsiveness	M5M total score (*n* = 5)	0	5	0	0	0	5	0	
	Positive factor (*n* = 5)	0	5	0	0	2	3	0	
	Negative factor (*n* = 5)	0	5	0	0	3	2	0	
	Disorganized thought factor (*n* = 5)	0	5	0	0	3	2	0	
	Uncontrolled hostility/ excitement (*n* = 5)	0	5	0	0	2	3	0	
	Anxiety/depression factor (*n* = 5)	0	5	0	0	2	3	0	

^*^ClinROM development rating resulted from *Criteria for content validity*.

^**^As the M5M is derived from the original three-subscale PANSS, the ClinROM development rating is identical to its ClinROM development. ^a^ “Sufficient”. ^b^ “Insufficient”. ^c^ “Indeterminate”. ^d^ “Inconsistent”.

*ClinROM development* (domain (D1) and *content validity* (D2) of the PANSS in its original three-subscale structure has been evaluated by us in another publication^7^. As the M5M emerged from the original three-subscale model^2^, no further *ClinROM development* and *content validity* studies were available.

In the 90 publications 128 suitable assessments (called ‘studies’ by COSMIN) of the *structural validity* (D3) for evaluating the M5M, were identified^24–97^. Most of them (64.84%) received “adequate” or “very good” ratings. Additionally, for each of the five factors, three analyses assessed the factors dimensionality^71^. All those dimensionality assessments received “adequate” ratings. Two assessments conducted confirmatory factor analyses on the negative factor^98^ and received “very good” ratings.

No analyses on the measurement properties *internal consistency (D4)*, *cross-cultural validity/ measurement invariance (D5)*, *reliability* (D6), measurement error (D7) and *criterion validity (D8)* could be identified for the M5M-PANSS^5^ and its factors.

Only five analyses for *hypothesis testing for construct validity* (D9) of the M5M total score were identified^99^ and all of them were rated “doubtful”. More analyses were available for the individual factors^99–101^, but most received “doubtful” ratings (70.59–100%), with a few analyses for the positive and the negative factor receiving “adequate” or “very good” ratings.

For the M5M total score and for each of the five factors, five analyses on responsiveness (D10) were identified^87^. All analyses on responsiveness received “adequate” ratings.

### Assessing the criteria for content validity and the updated criteria for good measurement properties

#### Assessing the criteria for content validity

No study matching COSMINs definition of a *ClinROM development* study was available for the M5M. Furthermore, as for the original three-subscale version^7^, there were no specific *content validity* assessments of the M5M available. Nevertheless, the domain *content validity* is independent of scale structure and therefore the *content validity* rating of the original three-subscale PANSS-30 was applicable to the M5M as well. Based on the original PANSS’ *ClinROM development* study^2^ and reviewer rating, the *content validity* rating was “inconsistent”^7^.

#### Assessing the updated criteria for good measurement properties

All results on the *updated criteria for good measurement properties* are reported in Table 2.

##### Structural validity (D3)

95 exploratory factor analyses,^92–95^ and 10 confirmatory factor analyses specifically of the M5M,^24,45,55,72,74^ for which fit indices were presented were identified. Only 12^5,28,31,43,46,49,58,63,68,70^ of the 95 exploratory derived models received “sufficient” ratings, 80 models^69,70,72,74,76,77,92–94^ received “insufficient” ratings, and 3 models^73,75,95^ received an “indeterminate” rating. CFI-values ranged between 0.511 and 0.887 and none surpassed COSMINs cut-off of CFI > 0.95 (or of ≥0.90 in the more relaxed sensitivity analysis), therefore those analyses were rated “insufficient”, as well. 67 of the 95 (70.5%) exploratory analyses resulted in five-factor solutions^5,54,56,58,59,61,79,82,83,85,88,94,95^. Furthermore, 9 exploratory analyses^3,34,57,58,62,72,92,93^ found four-factor models, 6^53,63,70,81,94^ found six-factor models, 7^46,81,87^ found seven-factor models, 2^80,84^ found bifactorial models (containing 1 general factor and 5 specific factors), 2^33,86^ found three-factor models, one^4^ found an eight-factor model, one^27^ found a nine-factor model.

23 further IRT-analytical analyses were identified^60,66,67,71,78,80,96,97^. 17 of the 23 analyses assessed the scalability of the PANSS^78,96^, of which 15 concluded that the PANSS is not scalable^78,96^, thus supporting the evidence indicating the insufficiency of the *structural validity* of the PANSS as a 30-item instrument, as reported in our previous publication^7^. Due to not fulfilling all *updated criteria* for IRT analyses^8–10^, all 23 analyses were rated “indeterminate”. Overall, the structural validity of the M5M^5^ was rated “insufficient”.

All principal component analyses assessing the dimensionality of the factors^71^ concluded that the M5M factors are unidimensional but were rated “indeterminate” due to not fulfilling all necessary *updated criteria*. Confirmatory factor analyses were only available for the negative factor. In 2 CFAs of the negative factor CFIs were 0.973 and 0.982^98^ surpassing COSMINs criterion of CFI > 0.95^8–10^ and were thus rated as “sufficient”.

Thus, we concluded that the structure of the M5M^5^ with 30 items is not satisfactory, but previous studies have still established that the factors are unidimensional and that the negative factor has a good model fit.

No analyses on the measurement properties *internal consistency (D4), cross-cultural validity*/ *measurement invariance* (D5), *reliability* (D6), *measurement error* (D7) and *criterion validity* (D8) of the M5M total score or the five factors could be identified.

##### Construct validity (D9)

5 analyses for hypothesis testing for *construct validity* with correlations between the M5M total score and its factors were identified^99^. The correlations ranged between 0.64 and 0.89, while we hypothesized that they would be lower, resulting in “insufficient” ratings.

12 to 17 analyses for hypothesis testing for *construct validity* of the M5M factors were identified^99–101^ with correlations of the factors with each other, the M5M total score, the CGI-S^102^ and Andreasen’s SAPS^103^ and SANS^104^. 70.59–83.33% of the assigned hypotheses could be confirmed for the respective factors, which were consequently rated “sufficient” for *construct validity*. An overview of the correlations and the respective hypotheses is presented in Appendix 5.

##### Responsiveness (D10)

5 analyses for the assessment of *responsiveness* were identified for the M5M total score and all 5 factors^87^. 40–60% of the hypotheses could be confirmed for the factors resulting in “inconsistent” ratings. None of the hypotheses for the M5M total scores could be confirmed hence why the *responsiveness* of it was rated “insufficient”. Nevertheless, the M5M has frequently and successfully been used in antipsychotic drug trials which we do not address here. Those trials indicate satisfactory responsiveness.

### GRADE approach

The quality of evidence regarding the *construct validity* of the M5M total score and of all factors but the negative factor was downgraded by one level to “moderate” quality of evidence due to a lack of well-executed analyses or inconsistent results.

The quality of evidence was not determined for the *responsiveness* of the five factors because they received inconsistent ratings in the updated criteria and were therefore not graded^8–10^.

The quality of all other measurement properties of the scale or the factors was not downgraded (compare Table 3).

**Table 3:** COSMIN Summary of findings.

	Summary or pooled result	Overall rating	Quality of evidence
*Content validity*^a^			
M5M total score	Relevance rating: ±, Comprehensiveness rating: −, Comprehensibility rating: ± (overall ratings resulting from the PROM development study and the reviewer rating)	Inconsistent	Low (no content validity analyses available)
*Structural validity*			
M5M total score	12/93 of the EFA derived models fulfill the criteria; CFI/NFI-range: 0.511–0.887 (total sample size: 12,025), 15/17 scalability assessments reject scalability	Insufficient	High (multiple analyses of very good quality; consistent results)
Positive factor	Unidimensional score	Indeterminate (3× (?))	High (multiple analyses of adequate quality; consistent results)
Negative factor	Unidimensional score; CFI-range: 0.973–0.982 (total sample size: 138)	Sufficient	High (multiple analyses of very good quality; consistent results)
Disorganized thought factor	Unidimensional score	Indeterminate (3× (?))	High (multiple analyses of adequate quality; consistent results)
Uncontrolled hostility/excitement factor	Unidimensional score	Indeterminate (3× (?))	High (multiple analyses of adequate quality; consistent results)
Anxiety/depression factor	Unidimensional score	Indeterminate (3× (?))	High (multiple analyses of adequate quality; consistent results)
*Hypothesis testing*			
M5M total score	0 out of 5 hypotheses confirmed	Insufficient	Moderate (multiple analyses of doubtful quality, consistent results)
Positive factor	12 out of 17 hypotheses confirmed	Sufficient	Moderate (multiple very good analyses, inconsistent results)
Negative factor	13 out of 17 hypotheses confirmed	Sufficient	High (multiple very good analyses, consistent results)
Disorganized thought factor	9 out of 12 hypotheses confirmed	Sufficient	Moderate (multiple analyses of doubtful quality, consistent results)
Uncontrolled hostility/excitement factor	9 out of 12 hypotheses confirmed	Sufficient	Moderate (multiple analyses of doubtful quality, consistent results)
Anxiety/depression factor	10 out of 12 hypotheses confirmed	Sufficient	Moderate (multiple analyses of doubtful quality, consistent results)
*Responsiveness*			
M5M total score	0 out of 5 hypotheses confirmed	Insufficient	High (multiple adequate analyses, consistent results)
Positive factor	2 out of 5 hypotheses confirmed	Inconsistent (2x (+), 3× (−); 60% (−))	None
Negative factor	3 out of 5 hypotheses confirmed	Inconsistent (3 (+), 2 (−); 60% (+))	None
Disorganized thought factor	3 out of 5 hypotheses confirmed	Inconsistent (3 (+), 2 (−); 60% (+))	None
Uncontrolled hostility/excitement factor	2 out of 5 hypotheses confirmed	Inconsistent (2x (+), 3× (−); 60% (−))	None
Anxiety/depression factor	2 out of 5 hypotheses confirmed	Inconsistent (2× (+), 3× (−); 60% (−))	None

^a^The domain content validity is independent of scale structure and therefore the content validity rating of the original three-subscale PANSS-30 was applicable to the M5M.

### Meta-analysis

Refer to Appendix 6 for pooled results and a more comprehensive overview of the data of individual analyses entering meta-analysis, all pooled results, as well as a forest plot of all correlations for *construct validity* and their 95% confidence interval. Publication bias could not be assessed because for no measurement property at least 10 analyses that could be pooled were available.

### Recommendation

Due to the high-quality evidence for “insufficient” *structural validity* of the M5M-PANSS it is categorized as (C), not recommendable, following the COSMIN guideline^8–10^.

### Feasibility

We gathered information on the feasibility of the PANSS in our publication on the original three-subscale PANSS^7^, which is applicable independent of the used model. Strengths of the PANSS are its availability in over 40 languages, simple score calculation by summing up item scores, the availability of the PANSS manual with its precise definitions, the Structured Clinicial Interview for PANSS assessments (SCI-PANSS)^105^ and the Informant Questionnaire (IQ-PANSS)^106^. Weaknesses are the scale’s long completion time of 30–50 min^2^, requirements for rater training to achieve acceptable interrater reliability making the standardization process laborious^107^, and the high cost of the instrument ($96.40 for the PANSS Technical Manual, $90.80 for 25 PANSS Rating and Profile Forms)^108^ due to copyright reasons. The European Medicines Agency (EMA)^109^ requires the use of the PANSS for drug approval. All the information gathered on feasibility as required by COSMIN can be found in Appendix 7.

### Assessment of fit indices of other five-factor models

As the M5M never met COSMIN’s criteria for fit indices, we examined other factor solutions, as well. 23 studies^1,24,41,45,53,55,56,72,74,80,84,85,97,110–119^ presented goodness of fit indices for other models than the M5M^5^ and the original Kay et al. structure^2^. A total of 73 models (52 five-factor, 6 six-factor, 4 four-factor, 3 three-factor, 2 two-factor, 2 unidimensional, 2 specific bifactorial models (containing 1 general factor and 5 specific factors), one seven-factor model, one eight-factor model) were assessed by 237 confirmatory analyses (the higher number of analyses than studies stems from the fact that most studies tested several models and several tested them in more than one sample).

The vast majority of these analyses (199)^1,24,41,45,55,56,72,74,80,84,85,97,111–119^ were on 5-factor models other than the M5M. The remaining analyses were 16^24,45,72,85,110,112,115^ on 4-factor models, 6^45,53,72,84,115^ on 6-factor models, 4^110–112^ on 3-factor models, 4^110–112^ on 2-factor models, 3^80,84^ on specific bifactorial-models (containing 1 general factor and 5 specific factors), 3^110,112^ on unidimensional models, one^41^ on a 7-factor model and one^72^ on an 8-factor model.

Only 19 of the 237 fit indices surpassed the COSMIN *updated criteria* criterion of CFI > 0.95/ RMSEA < 0.06. The respective fit indices belonged to an overall number of 9 models: the Wallwork^1^ five-factor model^85,119^, the Lindenmayer^49^ five-factor model^85^, 1 of 2 van der Gaag^56^ five-factor models^85^, the White^120^ five-factor model^85^, the Jiang^24^ five-factor model^24^, Freitas’ 2019^85^ five-factor model^85^, Kay and Sevys´^3^ pyramidical four-factor model^85^, Andersons´^80^ bifactorial model^80^, the Reichenberg^53^ six-factor model^53^. It should be noted that most of the CFIs with values > 0.95 were presented in Freitas et al. 2019^85^ (73.68%).

29 further fit indices on 22 models surpassed the criterion of CFI > 0.9 or RMSEA ≤ 0.08. In addition to the models just mentioned there were, the Strauss-Peralta 1974/1992^121,122^ four-factor model described by Cuesta and Peralta^110^, the Peralta^122^ three-factor model described by Cuesta and Peralta^110^, the Peralta and Cuesta^4^ four-factor model^110^, the Mass^42^ five-factor model^24^, the Lancon 2000^40^ five-factor model^24^, the Hayashi^113^ five-factor model^113^, the Fitzgerald^114^ five-factor model^24^, the Drake^45^ (I) five-factor model^45^, the Drake^45^ (II) five-factor model^45^, the Reichenberg^53^ six-factor model^53^, the van den Oord^115^ five-factor model^115^, the van den Oord^115^ six-factor model^115^ and Andersons´^80^ five-factor model^80^.

Due to the predominance of five-factor models in this research (identified in 67^5,54,56,58,59,61,79,82,83,85,88,94,95^/95^92–95^=71% of exploratory factor analyses, 6^1,24,49,56,85,120^/9^1,3,24,49,53,56,80,85,120^ =67% of those with COSMIN CFI > 0.95 and 14^1,24,40,42,45,49,56,80,85,120^/22^1,3,4,24,40,42,45,49,53,56,80,85,110,120–122^=64% of those with CFI ≥ 0.90) we further focused on those. They are presented in Table 4. Details on all models and fit indices are presented in Appendix 8.

**Table 4:**
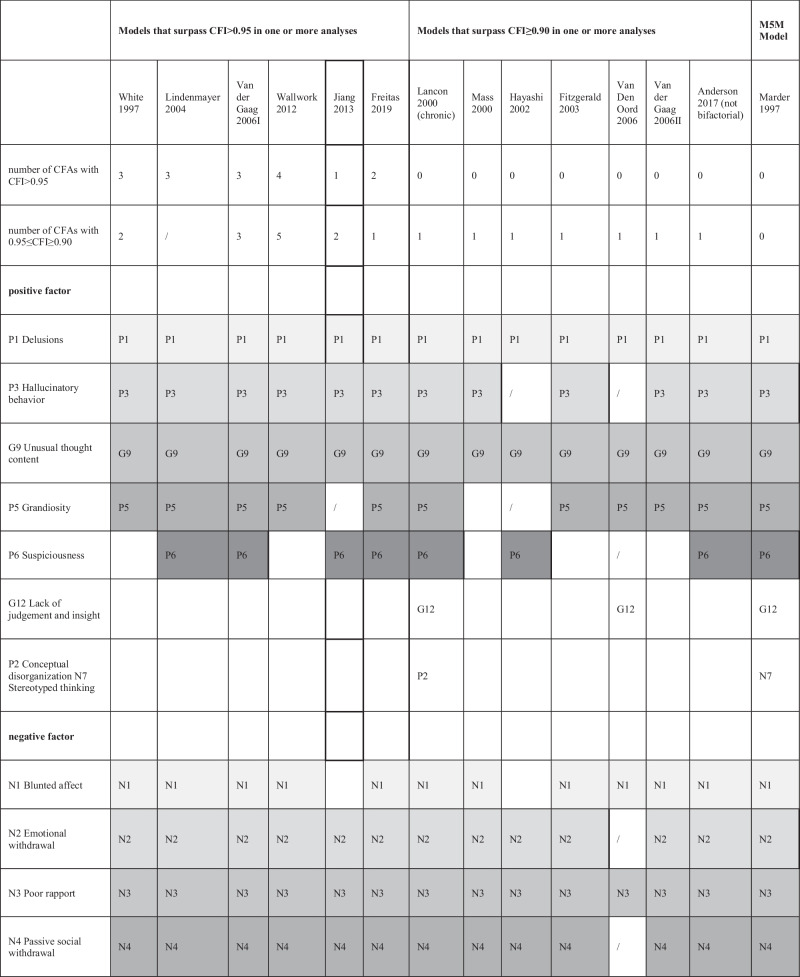

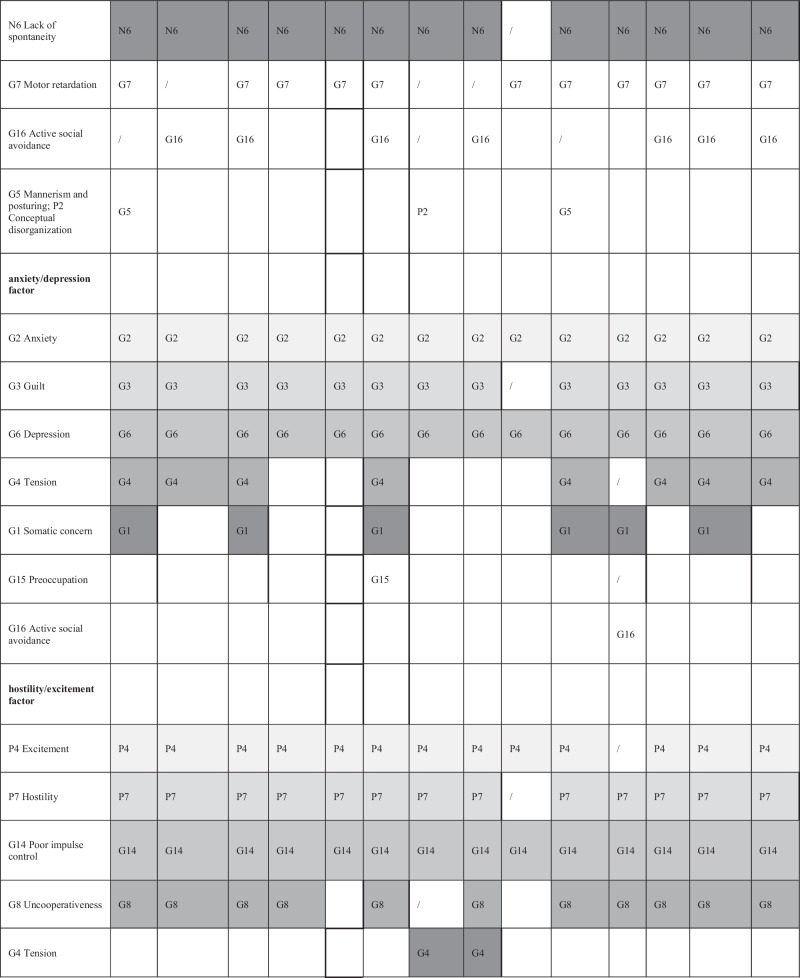

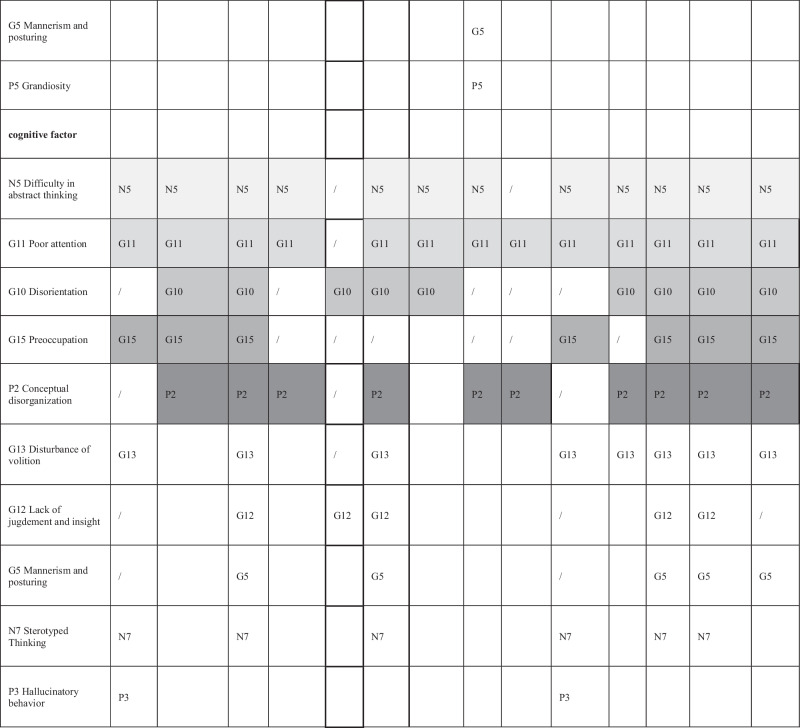
Item distribution of five-factor models^*^.

Item definitions in the left column are for understanding purposes only and correspond to all items that are assigned to the factor in at least one model. The use of grayscale is solely for simplified presentation and easier understanding of the table. We could not assess Drakes’ five-factor model^45^ further, as we were unable to obtain the exact factor structure or the information for which model the fit index was calculated from the article’s authors. There the M5M is presented, as well, for comparison, although it never reached an appropriate goodness of fit.

There were differences in frequency as to how often the 13 models had a CFI > 0.95/ ≥ 0.90: Wallwork (2012) had most (4^85,119^/9^1,24,85,117,119^); followed by White (1997) (3^85^/5^24,85^); Van der Gaag (2006I) (3^85^/6^24,56,85^); Lindenmayer (2004) (3^85^/3^85^); Freitas (2019) (2^85^/3^85^); Jiang (2013) (1^24^/3^24^). While the Wallwork^1^ model had the most satisfying fit indices and van der Gaags ranked second, they were as well among the most assessed models.

Several items seem to reliably load onto the same dimension in all five factor models which surpassed CFI > 0.95 at least once: P1 (Delusions), P3 (Hallucinatory behavior) and G9 (Unusual thought content) on the positive factor, N2 (Emotional withdrawal), N3 (Poor rapport), N4 (Passive social withdrawal) and N6(Lack of spontaneity) on the negative factor, G2 (Anxiety), G3 (Guilt) and G6(Depression) on the anxiety/depression factor, P4 (Excitement), P7 (Hostility) and G14 (Poor impulse control) on the hostility/excitement factor.

Several items seem to reliably load onto the same dimension in all five-factor models which surpassed CFI ≥ 0.90 at least once: P1 and G9 on the positive factor, N3 on the negative factor, G2 and G6 on the anxiety/ depression factor, G14 on the hostility/ excitement factor. Notably, the same consistencies in item distribution also apply to the M5M model. No items could be identified which consistently loaded on the cognitive factor in all of these models, and the composition of this factor was least homogeneous.

## Discussion

The M5M-PANSS^5^ is very frequently used in antipsychotic drug trials. This systematic review is, to the best of our knowledge, the first to assess the measurement properties of the M5M following COSMIN^8–10^. We included 95 publications and were only able to assess three COSMIN measurement properties, namely *structural validity*, *construct validity* and *responsiveness*, for the other seven domains no data were available. Good *construct validity* is a strength, because the domains correlated strongly with other rating scales on the same constructs. We already determined that the *content validity* of the PANSS has short-comings in our COSMIN review of the original three-subscale PANSS^7^. However, the main limitation of the M5M is *structural validity*, because goodness of fit measures did not reach COSMIN’s criterion of CFI > 0.95 and the more relaxed criterion of CFI ≥ 0.90. Some other 5-factor solutions had better fit indices.

Most psychometric analyses assessing PANSS factor solutions focused almost entirely on *structural validity*. It was found out relatively early that the original 3 subscale version is not appropriate^3^, hence researchers tried to identify factor structures with better fit. In doing so they overlooked that any new factor structure means that we are essentially talking about a new instrument with new subscales which require a full assessment of other measurement properties, as well^8–10^. One reason as to why this has so far not been properly done may be that to date no consistently fitting factor solution has been identified (see below). Nevertheless, such analyses would be important if the original PANSS were to be replaced by other factor solutions.

We could not confirm “sufficient” goodness of fit for the M5M solution. We, therefore, examined other solutions. In 66 publications 95 different factor-models were found by exploratory factor analyses and 73 were examined with confirmatory methods. Most of these yielded 5-factor solutions (67/95 models) so that we focused on them. Overall, their factor content was usually similar. Nevertheless, there occurred a lot of item changes between the factors which are probably caused by differences in populations (e.g. acutely ill versus stable state of disease or different diagnoses), differences in fit indices applied or sample size (leading to more or less uncertainty). Therefore, it was impossible for us to identify *the* best factor solution.

Our assessment of the PANSS factor structure shows that it is difficult to identify a uniform factor solution of the PANSS which provides good fit in different and heterogeneous populations. However, we also identified consistencies among the models. The description of which items went to which factor in the various models revealed a certain pattern (see Table 4). Some items always loaded on the same factors. Therefore, the names given to the identified factors were relatively consistent across models. We emphasize that the cognitive factor is the least robust factor in terms of item to factor assignment.

Two models stood out. Wallwork et al.^1^ identified a model solution by counting “votes” from published factor analyses which was subsequently tested and validated in several independent publications. Van der Gaag et al.^56^ used ten-fold cross-validation as a particularly stringent method. In our work both Wallwork’s and van der Gaag’s models most frequently had good fit indices. It should also be noted that the M5M, although it showed no good model fit, was relatively similar in item composition to the other models summarized in Table 4. Shafer et al. 2019^123^ conducted a meta-analysis on the structure of the PANSS and BPRS-E scales, identifying “core items” among them. Shafer’s’ findings are largely consistent with our results and should also be considered in the development process of scales based on the PANSS items.

## Limitations

Several limitations of this review, some of which are similar to those of our previous publication^7^, must be acknowledged. The first two limitations relate purely to the shortcomings of the COSMIN methodology, while the subsequent ones also contain matters relating to the psychometric literature on the PANSS.

First, our “vote count approach” in the comparison of models is certainly far from ideal, e.g. the COSMIN cutoff 0.95 for confirmatory factor analyses is to some extent arbitrary. However, our analysis with the frequently used cutoff in the literature 0.90 which is more relaxed yielded comparable results.

Second, while the COSMIN framework offers a structured approach to evaluate the measurement properties of instruments like the PANSS, it does not accommodate certain methodologies that might provide a more precise analysis. This required us to use certain workarounds to accommodate for the variety of approaches used and prevented us from assessing a few analyses like Network analyses^124^, orthonormal projective non-negative matrix factorization (OPNMF)^125^ leading to their exclusion and certain structural equation modeling (SEM) analyses^73,75^ which could not be qualitatively evaluated and were rated “indeterminate”^7^. Due to the shortcomings of COSMIN, several methodologically different procedures with different levels of validity were assessed based on the same criteria, e.g. no consideration of different rotation methods or eigenvalue criteria^7^. Nevertheless, the results of non-included or “indeterminate” rated assessments support our findings.

Third, most measurement properties of the M5M-PANSS have not been addressed yet, therefore, we cannot make a statement regarding them. Thus, it is possible that if future studies assessed them, our recommendation would become better.

Fourth, we focussed on the M5M, because it is frequently used in antipsychotic drug trials. Ideally, we would also have addressed all other solutions, but there are so many (95!) that this would not have been feasible. Moreover, as there is so little evidence on the M5M (we identified 4 validation studies), it is unlikely that the other solutions have been assessed more thoroughly.

Fifth, the PANSS is an instrument with ordinal scaling but, following the original authors of the included studies, we assumed that it is continuous^7^.

Sixth, differences in the PANSS assessments between analyses must be considered. The SCI-PANSS^105^, a structured clinical interview, and the IQ-PANSS^106^, a structured informant questionnaire, are available for PANSS assessments. Additionally, the PANSS Institute provides training for raters. However, the use of these instruments and the rater level are often not specified or may vary.

Seventh, 74% of the “sufficient” fit indices were extracted from the same publication^85^, in which 78% of all calculated fit indices for different models surpassed CFI ≥ 0.95. Models with reportedly good fit in the Freitas publication had worse fit in some other analyses (e.g. Wallwork-model’s CFI in Freitas et al. (2019)^85^=0.964, Wallwork-model’s CFI in Langeveld et al.^116^=0.812). Therefore, and because Freitas et al.’s analyses were conducted in samples of patients in a stable disease state^85^, these results must be treated with caution. The good model fit might not apply to other populations such as acutely ill patients or as differences might be due to differences in methodological approaches, clinical heterogeneity and interviewer variability across multiple centers. Only 4 of the previous 9 models achieve the COSMIN cutoff of CFI > 0.95, if the publication by Freitas (in the sense of a sensitivity analysis) is disregarded. These are the Jiang^24^ five-factor model, the Anderson^80^ bifactorial model, the Reichenberg^53^ six-factor model and the Wallwork^1^ five-factor model. The Wallwork^1^ five-factor model is the only one of these four that achieves CFI > 0.95 in more than 1 analysis. This suggests a certain independence of the positive results of the Wallwork^1^ model from the possible bias of the Freitas 2019^85^ publication.

Eight, the COSMIN criteria do not address the situation if items loaded on various factors which the original authors resolved by assigning such items to the factors they most strongly loaded on.

## Conclusion

We conclude that the Marder five-factor PANSS has “insufficient” structural validity, although most of the 5 factor solutions (including M5M) have core items in common across all factors except the cognitive factor. Therefore, future validation studies may focus on other models such as the 5-factor model proposed by Wallwork and colleagues. Any such model requires a full exploration of its measurement properties before it can be generally recommended.

## Supplementary information

Appendix files

## Data Availability

The publications from which the data were analyzed in the current study are available in the PubMed and Embase databases. PubMed can be accessed via [https://pubmed.ncbi.nlm.nih.gov], Embase is a subscription-based database that requires institutional or individual paid access. References to all included publications can be found in the reference section of this publication. The meta-analytical data generated during this study are available within the paper and its supplementary files. No specific data repository was used; therefore, accession codes are not needed.
