# Supplementary material for: COSMIN review of the PANSS Marder factor solution and other factor models in people with schizophrenia
Source: Schizophrenia (Heidelb). 2025 Mar 31;11(1):51. doi: 10.1038/s41537-025-00600-6 (PMC11958836; doi:10.1038/s41537-025-00600-6)
Supplement: Supplementary file 1 — Appendix files [file 41537_2025_600_MOESM1_ESM.pdf]

Supplementary content/ Appendix

Appendix1 - OSF Study protocol

Appendix2 - Search filters for EMBASE and MEDLINE

Appendix3 - PRISMA Diagram of the Search

Appendix4 - Characteristics of the included study populations

Appendix5 – Correlations for construct validity and respective hypotheses

Appendix6 - Meta-analytical results for convergent validity

Appendix7 – Information on feasability

Appendix8 - Confirmatory analyses of further PANSS models

This supplementary material has been provided by the authors to give readers additional information about their work.

Appendix1 - OSF Study Protocol

Systematic review of the Positive and Negative Syndrome Scale following the COSMIN standard

By Simon Geck, Maximilian Roithmeier, Stefan Leucht
Publication date: 2024-01-31
Publisher: Center for Open Science

Review question: How suitable are the Positive and Negative Syndrome Scale (PANSS), its Positive and Negative Subscores and short forms / patient-versions to evaluate/capture/document the symptom severity in patients with schizophrenia?

Search sources: MEDLINE via PubMED, EMBASE. Additionally, screening of the reference lists of included studies for further publications. No other sources including unpublished studies will be sought. No restrictions on publication period. Articles will be included independent of the language they are in. According to the COSMIN standard only full text articles will be included.

Search dates: from database inception until 09.10.2023.

Types of study to be included: Any study that reports on the measurement properties of PANSS- Versions (i.e. PANSS-total, PANSS-neg, PANSS-patient, PANSS-6) examined by us. Only published full-text peer-reviewed articles or manuals will be included.
Condition or domain being studied Symptom severity in terms of symptoms of schizophrenia in patients with schizophrenia spectrum disorders.

Participants/population: No restriction on age or phase of disease.

Assessed instruments: Clinician- or patient-reported rating scales that assess disease-related symptom severity, such as PANSS-total, PANSS-6, PANSS-patients.

Context: Studies that report on the development and/or psychometric evaluation of observer-reported/ self-reported measures that assess disease-specific severity of symptoms will be included. No restrictions regarding setting.

Procedure:
Following the COSMIN manual, the following steps described in said manual will be conducted by two reviewers (SG and MR respectively) independently:

Risk of bias assessment:
The methodological quality of individual studies will be assessed using the COSMIN Risk of Bias checklist (Mokkink et al., 2018) consisting of 10 boxes which consist of several standards. The checklist will be conducted for each publication separately. Only the corresponding boxes of psychometric properties evaluated by the respective publication will be evaluated. The following boxes are included in the COSMIN Risk of Bias checklist:
- Box 1. Standards for evaluating the quality of PROM development
- Box 2. Standards for evaluating the quality of content validity studies of PROMs
- Box 3. Structural validity

- Box 4. Internal consistency
- Box 5. Cross-cultural validity/ measurement invariance
- Box 6. Reliability
- Box 7. Measurement error
- Box 8. Criterion validity
- Box 9. Hypotheses testing for construct validity
- Box 10. Responsiveness

After independently conducting the ratings, both reviewers will try to find consensus on their ratings. If consensus cannot be reached, a third, professor level reviewer (SL) will be consulted.

Criteria for good measurement properties:
Following the Risk of bias assessment, the characteristics of the included study population as well as the characteristics of the included PROMs will be summarized in two tables. For each of the included references the characteristics of the included validation studies will be described as exemplary shown in Appendix 3 respectively Appendix 4 of the COSMIN manual, , i.e. construct(s), target population, mode of administration, case numbers, gender distribution, ae mean (SD, range), disease severity etc..
Afterwards the result of each study on a measurement property will be rated against the updated criteria for good measurement properties (Terwee et al., 2007). Hereby each result is to be evaluated as either sufficient (+), insufficient (-), inconsistent (#), or indeterminate (?) (Prinsen et al., 2018).

Summarization of evidence and grading of the evidences quality:
While the Risk of bias assessment and the updated criteria for good measurement properties focus on the quality of individual studies, afterwards the quality of the whole PROM/ ClinROM will be assessed. Therefor the evidence will be summarized by, if the data is consistent, quantitatively pooling the results. The pooled results will then, as described above for individual studies, be compared to the updated criteria for good measurement properties. The quality of the resulting evidence will then be graded as either high, moderate, low or very low according to a modified GRADE (Grades of Recommendation, Assessment, Development and Evaluation) approach (see GRADE Handbook, 2013).

Recommendations:
If more instruments would be included in a COSMIN review, recommendations on the most suitable one for use in evaluative application would be formulated. As we just assess the PANSS scale, said scale will just be assigned to a category from A to C.
PROMS(/ClinROMS) in category 'A' can be recommended for use and their results can be trusted. PROMs(/ClinROMs) categorized as ‘B’ have potential to be recommended for use, but further research is necessary to assess the quality of these instruments.
PROMS(/ClinROMS) in category 'C' can't be recommended for use.

As last step, our systematic review depicting our findings will be reported.

Appendix2 - Search filters for EMBASE and MEDLINE

EMBASE

1 "Positive and Negative Syndrome Scale"/ or (PANSS* or "Positive and Negative Symptom" or "Positive and Negative Symptoms" or "Positive and Negative Syndrome" or "Positive and Negative Syndromes").mp. (21483)
2 exp Schizophrenia Spectrum Disorder/ or exp Schizophrenia/ or Schiz*.mp. (277621)
4 exp intermethod comparison/ or exp data collection method/ or exp validation study/ or exp feasibility study/ or exp pilot study/ or exp psychometry/ or exp reproducibility/ or exp observer variation/ or exp discriminant analysis/ or exp validity/ or (reproducib* or audit or psychometr* or clinimetr* or clinometr* or observer variation or reliab* or valid* or coefficient or internal consistency or (cronbach* and (alpha or alphas)) or item correlation or item correlations or item selection or item selections or item reduction or item reductions or agreement or precision or imprecision or precise values or test-retest or (test and retest) or (reliab* and (test or retest)) or stability or interrater or inter-rater or intrarater or intra-rater or intertester or inter-tester or intratester or intratester or interobeserver or inter-observer or intraobserver or intraobserver or intertechnician or inter-technician or intratechnician or intratechnician or interexaminer or inter-examiner or intraexaminer or intraexaminer or interassay or inter-assay or intraassay or intra-assay or interindividual or inter-individual or intraindividual or intra-individual or interparticipant or inter-participant or intraparticipant or intraparticipant or kappa or kappas or coefficient of variation or repeatab* or ((replicab* or repeated) and (measure or measures or findings or result or results or test or tests)) or generaliza* or generalisa* or concordance or (intraclass and correlation*) or discriminative or known group or factor analysis or factor analyses or factor structure or factor structures or dimensionality or subscale* or multitrait scaling analysis or multitrait scaling analyses or item discriminant or interscale correlation or interscale correlations or ((error or errors) and (measure* or correlat* or evaluat* or accuracy or accurate or precision or mean)) or individual variability or interval variability or rate variability or variability analysis or (uncertainty and (measurement or measuring)) or standard error of measurement or sensitiv* or responsive* or (limit and detection) or minimal detectable concentration or interpretab* or (small* and (real or detectable) and (change or difference)) or meaningful change or minimal important change or minimal important difference or minimally important change or minimally important difference or minimal detectable change or minimal detectable difference or minimally detectable change or minimally detectable difference or minimal real change or minimal real difference or minimally real change or minimally real difference or ceiling effect or floor effect or item response model or irt or rasch or differential item functioning or dif or computer adaptive testing or item bank or cross-cultural equivalence).ti,ab. (8081187)

MEDLINE

1 exp Schizophrenia/ or Schiz*.mp. (197059)
2 (PANSS* or "Positive and Negative Symptom" or "Positive and Negative Symptoms" or "Positive and Negative Syndrome" or "Positive and Negative Syndromes").mp. (9644)
3 exp Psychometrics/ or exp Outcome Assessment, Health Care/ or exp Observer Variation/ or exp Health Status Indicators/ or exp Reproducibility of Results/ or exp Discriminant Analysis/ or (instrumentation or methods).sh. or (validation study or comparative study).pt. or (clinimetr* or clinometr* or outcome measure* or agreement or precision or imprecision or precise values

or repeatab* or ((replicab* or repeated) and (measure or measures or findings or result or results or test or tests))).mp. or (psychometr* or outcome assessment or observer variation or reproducib* or reliab* or unreliab* or valid* or coefficient of variation or coefficient or homogeneity or homogeneous or internal consistency or (cronbach* and (alpha or alphas)) or (item and (correlation* or selection* or reduction*)) or test-retest or (test and retest) or (reliab* and (test or retest)) or stability or interrater or inter-rater or intrarater or intra-rater or intertester or inter-tester or intratester or intra-tester or interobserver or inter-observer or intraobserver or intra-observer or intertechnician or inter-technician or intratechnician or intra-technician or interexaminer or inter-examiner or intraexaminer or intra-examiner or interassay or inter-assay or intraassay or intra-assay or interindividual or inter-individual or intraindividual or intra-individual or interparticipant or inter-participant or intraparticipant or intra-participant or kappa or kappas or generaliza* or generalisa* or concordance or (intraclass and correlation*) or discriminative or known group or factor analysis or factor analyses or factor structure or factor structures or dimension* or subscale* or (multitrait and scaling and (analysis or analyses)) or item discriminant or interscale correlation* or (error or errors) or individual variability or interval variability or rate variability or (variability and (analysis or values)) or (uncertainty and (measurement or measuring)) or standard error of measurement or sensitiv* or responsive* or (limit and detection) or minimal detectable concentration or interpretab* or ((minimal or minimally or clinical or clinically) and (important or significant or detectable) and (change or difference)) or (small* and (real or detectable) and (change or difference)) or meaningful change or ceiling effect or floor effect or item response model or IRT or Rasch or differential item functioning or DIF or computer adaptive testing or item bank or cross-cultural equivalence).tw. (9172531)

Appendix3 – PRISMA diagram of the search

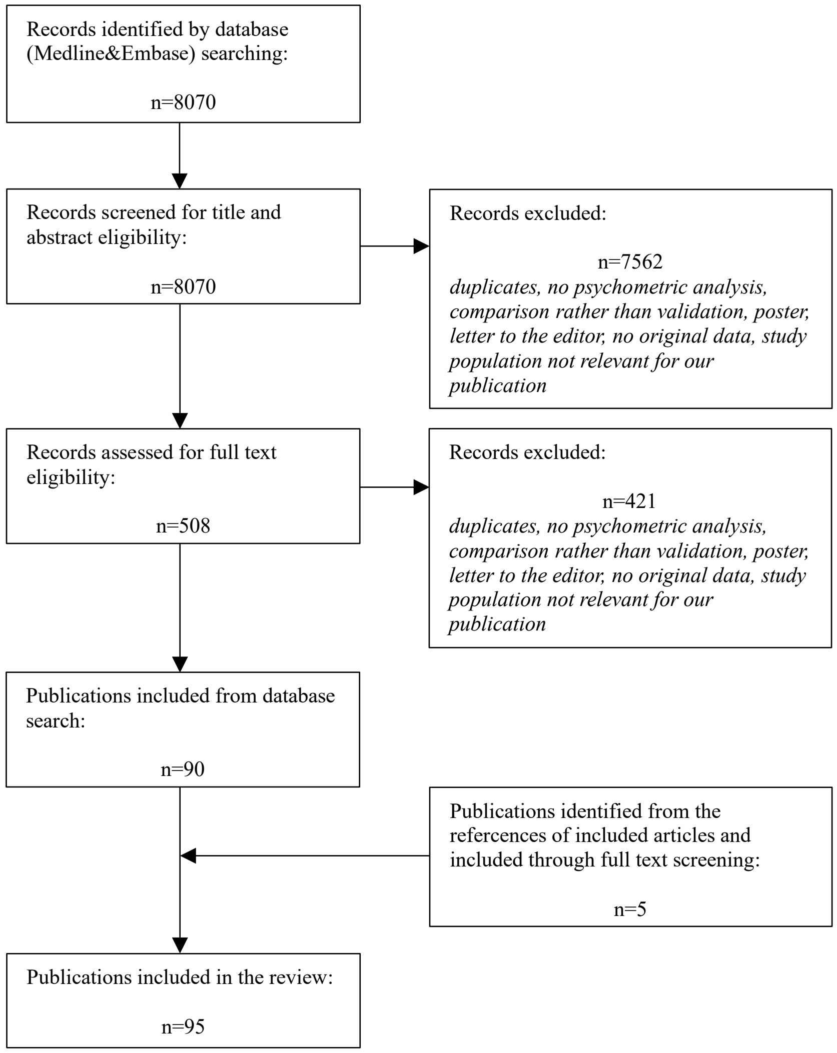

Appendix4 - Charcteristics of the included study populations								
		Population		Disease characteristics		Instrument administration		
Ref	N	Age mean	Gender %female	Disease	Disease duration mean (SD) yr	Setting	Country	Language
Dollfus, S., et al. (1991). "Principal-component analysis of PANSS and SANS-SAPS global ratings in schizophrenic patients." European Psychiatry 6(5): 251-259.	70	40.7 (SD = 14.3)	54.3%	patients with diagnosis of schizophrenia according to different diagnosis systems (DSM-3-R; Langfeldt; Carpenter; Schneider)	6.6 (SD = 5)	In- and Outpatients; Hospital Charles Nicolle, Rouen, France	France	French
Bell, M. D. (1994). "Five-Component model of schizophrenia: Assessing the factorial invariance of the positive and negative syndrome scale." Psychiatry Research 52(3): 295-303.	146	40 (SD = 8.3)	5%	patients meeting DSM-III-R criteria for diagnosis of chronic schizophrenia	16.6 (SD = 8.3)	Outpatients; VA Medical Center West Heaven, CT	USA	English
Peralta, V. (1994). "Psychometric properties of the positive and negative syndrome scale (PANSS) in schizophrenia." Psychiatry Research 53(1): 31-40.	100	35.8 (SD=12.7)	31%	patients meeting DSM-III-R criteria for diagnosis of schizophrenia	10.4 (SD = 8.9)	Inpatients	Spain	Spanish
	27	NIP on Age	NIP on Gender	patients meeting DSM-III-R criteria for diagnosis of schizophrenia	NIP on disease duration	Inpatients	Spain	Spanish
Dollfus, S. and M. Petit (1995). "Principal-component analyses of PANSS and SANS-SAPS in schizophrenia: their stability in an acute phase." Eur Psychiatry 10(2): 97-106.	57	40.3 (SD = 13.9)	66.7%	patients diagnosed with schizophrenia according to different diagnosis systems (DSM-3-R; Langfeldt; Carpenter; Schneider)	NIP on disease duration	Inpatients; Hospital Charles Nicolle, Rouen, France	France	French
Lançon, C., et al. (1998). "Factorial structure of the Positive and Negative Syndrome Scale (PANSS): a forced five-dimensional factor analysis." Acta Psychiatr Scand 98(5): 369-376.	205	36.2 (SD = 10.4)	40%	patients meeting DSM-III-R criteria for diagnosis of schizophrenia	11.2 (SD = 8.4)	In-patients and out-patients	France	French
Lançon, C., et al. (1999). "Validity and reliability of the French-language version of the Positive and Negative Syndrome Scale (PANSS)." Acta Psychiatr Scand 100(3): 237-243.	342	36.8 (SD = 10.91)	40.35%	patients meeting DSM-III-R criteria for diagnosis of schizophrenia	13.2 (SD = 9.4)	In- and Outpatients	France	French

		Population		Disease characteristics		Instrument administration		
**Ref**	**N**	**Age mean**	**Gender %female**	**Disease**	**Disease duration mean (SD) yr**	**Setting**	**Country**	**Language**
Lançon, C., et al. (2000). "Stability of the five-factor structure of the Positive and Negative Syndrome Scale (PANSS)." Schizophr Res 42(3): 231-239.	342	36.8 (SD = 10.91)	40.35%	patients meeting DSM-III-R criteria for diagnosis of schizophrenia in relapse and chronic phase	13.2 (SD = 9.4)	In- and Outpatients	France	French
Mass, R., et al. (2000). "Psychopathological syndromes of schizophrenia: evaluation of the dimensional structure of the positive and negative syndrome scale." Schizophr Bull 26(1): 167-177.	253	35.2 (SD = 10.6)	15.4%	patients meeting ICD-10 criteria for diagnosis of schizophrenia	7.6 (SD = 8.6)	Inpatients; Northern Clinic, Hamburg-Ochsenzoll & University Hospital, Hamburg-Eppendorf	Germany	German
Wolthaus, J. E. D., et al. (2000). "Component structure of the Positive And Negative Syndrome Scale (PANSS) in patients with recent-onset schizophrenia and spectrum disorders." Psychopharmacology 150(4): 399-403.	138	23.2 (SD = 5.26)	23.19%	first or second episode DSM-IV diagnosed schizophrenia	NIP on disease duration	In- and Outpatients; Universtiy Hospital Amsterdam, Groningen and Utrecht	Netherlands	Dutch
El Yazaji, M., et al. (2002). "Validity of the depressive dimension extracted from principal component analysis of the PANSS in drug-free patients with schizophrenia." Schizophr Res 56(1-2): 121-127.	81	30.42 (SD = 6.83)	12.36%	patients meeting DSM-IV criteria for diagnosis of acute schizophrenia	5.75 (SD = 4.65)	Inpatients; Centre Psychiatrique Universitaire of Casablanca	Morocco	French
Emsley, R., et al. (2003). "The factor structure for the Positive and Negative Syndrome Scale (PANSS) in recent-onset psychosis." Schizophrenia Research 61(1): 47-57.	535	26 (SD = 6.9)	28.97%	patients meeting DSM-IV criteria for diagnosis of schizophrenia, schizophreniform disorder or schizoaffective disorder	11.9 (SD = 3.11)	No single parameter on disease severity	11 Different countries	NIP (assumable English)

		Population		Disease characteristics		Instrument administration		
**Ref**	**N**	**Age mean**	**Gender %female**	**Disease**	**Disease duration mean (SD) yr**	**Setting**	**Country**	**Language**
Fitzgerald, P. B., et al. (2003). "A confirmatory factor analytic evaluation of the pentagonal PANSS model." Schizophr Res 61(1): 97-104.	347	33.8 (SD = 1.9)	38.04%	patients with schizophrenia/ schizoaffective disorder; no diagnostic system given	NIP on disease duration	Inpatients (48.7%) and outpatients (51.3%); Dandenong Psychiatry Research center, Victoria, AU	Australien	English
Loas, G., et al. (1997). "A four-syndrome model of chronic schizophrenia: Principal components analysis of the Postive and Negative Syndrome Scale (PANSS) in 153 chronic schizophrenics." Encephale 23(1): 10-18.	153	38.83 (SD = 10.15	41.83%	First or second episode DSM-IV diagnosed schizophrenia	14.69 (SD = 9.64)	In- and Outpatients; Hopital psychiatrique de Clermont de l´Oise and Hopital Pinel d´Amiens)	France	French
Lancon, C., et al. (1997). "Psychometric properties of the PANSS in a sample of French schizophrenic patients." Encephale 23(1): 1-9.	85	36.2 (SD = 11.3)	29.4%	patients meeting DSM-III-R criteria for diagnosis of schizophrenia	12 (SD = 9.08)	In- and Outpatients; Marseille	France	French
Fresán, A., et al. (2005). "A forced five-dimensional factor analysis and concurrent validity of the Positive and Negative Syndrome Scale in Mexican schizophrenic patients." Schizophr Res 72(2-3): 123-129.	150	29.5 (SD = 8.1)	38%	patients meeting DSM-IV criteria for diagnosis of schizophrenia	NIP on disease duration	In- and Outpatients (85.3% outpatients, 14.7% inpatients); National Institute of Psychiatry; Mexico City	Mexico	NIP (assumable Spanish)

		Population		Disease characteristics		Instrument administration		
**Ref**	**N**	**Age mean**	**Gender %female**	**Disease**	**Disease duration mean (SD) yr**	**Setting**	**Country**	**Language**
Van Den Oord, E. J. C. G., et al. (2006). "Factor structure and external validity of the PANSS revisited." Schizophrenia Research 82(2-3): 213-223.	499	38.37 (SD = 11.41)	35.3%	patients meeting DSM- IV or ICD-10 criteria for diagnosis of schizophrenia	NIP on disease duration	In- and Outpatients; Munich area, Germany	Germany	NIP (assumable German)
Van der Gaag, M., et al. (2006). "The five-factor model of the Positive and Negative Syndrome Scale I: confirmatory factor analysis fails to confirm 25 published five-factor solutions." Schizophr Res 85(1-3): 273-279.	5769	36.11 (SD = 11.42)	30.5%	patients meeting DSM-IV criteria for diagnosis of schizophrenia and related diseases	12.74 (SD =10.11)	In- and Outpatients	International collected data	several languages
Van der Gaag, M., et al. (2006). "The five-factor model of the Positive and Negative Syndrome Scale II: a ten-fold cross-validation of a revised model." Schizophr Res 85(1-3): 280-287	5769	36.11 (SD = 11.42)	30.5%	patients meeting DSM-IV criteria for diagnosis of schizophrenia and related diseases	12.74 (SD =10.11)	In- and Outpatients	International collected data	several languages
Masiak, M. and B. Loza (2004). "Core factors of schizophrenia structure based on PANSS and SAPS/SANS results. Discerning and head-to-head comparison of PANSS and SASPS/SANS validity." Psychiatria Polska 38(5): 795-808.	100	33.5 (SD = 7.95	36%	patients meeting ICD-10-DCR criteria for diagnosis of paranoid schizophrenia with current exacerbation	7.93 (SD = 6.68)	NIP	Poland	NIP (assumable Polish)
Levine, S. Z. and J. Rabinowitz (2007). "Revisiting the 5 dimensions of the Positive and Negative Syndrome Scale." J Clin Psychopharmacol 27(5): 431-436.	1872	NIP on Age	29.4%	chronic schizophrenia, schizophreniform disorder, schizoaffective disorder	NIP on disease duration	NIP	17 different countries	NIP
	1284	NIP on Age	29.4%	chronic schizophrenia, schizophreniform disorder, schizoaffective disorder	NIP on disease duration	NIP	17 different countries	NIP
Santor, D. A., et al. (2007). "Item response analysis of the Positive and Negative Syndrome Scale." BMC Psychiatry 7: 66.	9205	39.0 (SD = 11.5)	35%	patients meeting DSM-IV criteria for diagnosis of schizophrenia, schizoaffective, schizophreniform disorder	NIP on disease duration	In- and Outpatients	International collected data	NIP (assumable several languages)
Lindenmayer, J. P., et al. (2008). "Dimensions of psychosis in patients with bipolar mania as measured by the positive and negative syndrome scale." Psychopathology 41(4): 264-270.	271	NIP on Age	NIP on Gender	patients meeting DSM-IV criteria for diagnosis of bipolar disorder I with mania and psychotic symptoms (subpopulation of DSM-IV diagnosis of bipolar disorder I with mania)	NIP on disease duration	NIP	USA& India	English, Indian

		Population		Disease characteristics		Instrument administration		
**Ref**	**N**	**Age mean**	**Gender %female**	**Disease**	**Disease duration mean (SD) yr**	**Setting**	**Country**	**Language**
Gil, D., et al. (2009). "Validity of the cognitive factor of the Positive and Negative Syndrome Scale as a measure of cognitive functioning in schizophrenia." Rev Psiquiatr Salud Ment 2(4): 160-168.	235	NIP on age mean	37.02%	patients meeting ICD-10 criteria for diagnosis of schizophrenia	NIP on disease duration	CRPS of Padre Menni Hospital Center, Santander	Spain	Spanish
Citrome, L., et al. (2011). "Efficacy of iloperidone in schizophrenia: a PANSS five-factor analysis." Schizophr Res 131(1-3): 75-81.	3580	NIP on Age	NIP on Gender	patients meeting DSM-IV criteria for diagnosis of schizophrenia or schizoaffective disorder	NIP on disease duration	NIP	International	NIP
Khan, A., et al. (2011). "Use of non-parametric item response theory to develop a shortened version of the Positive and Negative Syndrome Scale (PANSS)." BMC Psychiatry 11: 178.	7348	NIP on Age	NIP on Gender	patients meeting DSM-IV criteria for diagnosis of schizophrenia or schizoaffective disorder	NIP on disease duration	NIP; Data provided by Ortho-McNeil Janssen Pharmaceutics	NIP	NIP
Wallwork, R. S., et al. (2012). "Searching for a consensus five-factor model of the Positive and Negative Syndrome Scale for schizophrenia." Schizophr Res 137(1-3): 246-250.	458	34.8 (SD = 9.9)	25%	patients meeting DSM-IV criteria for diagnosis of schizophrenia or schizoaffektive disorder	NIP on disease duration	NIP	USA	English
	164	37.4 (SD = 11.5)	45%	patients meeting DSM-IV criteria for diagnosis of schizophrenia	NIP on disease duration	NIP	Japan	Japanese
Kumar, A. and C. R. J. Khess (2012). "Factor analysis of positive and negative syndrome scale in schizophrenia: An exploratory study." Indian Journal of Psychiatry 54(3): 233-238.	150	32.97 (SD = 8.02)	33.4%	patients meeting ICD-10 criteria for diagnosis of schizophrenia	5.52 (SD = 4.69)	Inpatients; Central Institute of Psychiatry, Ranchi	India	Indian
Jiang, J., et al. (2013). "Validated five-factor model of positive and negative syndrome scale for schizophrenia in Chinese population." Schizophr Res 143(1): 38-43.	903	49.6 (SD = 13.0)	33.11%	patients meeting DSM-IV criteria for diagnosis of schizophrenia	21.8 (SD = 13.0)	NIP; Institute of Mental Health of Singapore	China	NIP (assumable Chinese)
	942	41.5 (SD = 10.8)	46.07%	patients meeting DSM-IV criteria for diagnosis of schizophrenia	17.4 (SD = 11.1)	NIP; Institute of Mental Health of Singapore	China	NIP (assumable Chinese)

		Population		Disease characteristics		Instrument administration		
**Ref**	**N**	**Age mean**	**Gender %female**	**Disease**	**Disease duration mean (SD) yr**	**Setting**	**Country**	**Language**
	1845	45.5 (SD = 12.6)	39.72%	patients meeting DSM-IV criteria for diagnosis of schizophrenia	19.6 (SD = 12.3)	NIP; Institute of Mental Health of Singapore	China	NIP (assumable Chinese)
Hwang, S. S. H., et al. (2009). "The causal model of insight in schizophrenia based on the positive and negative syndrome scale factors and the structural equation modeling." Journal of Nervous and Mental Disease 197(2): 79-84.	342	33.02 (SD = 9.07)	51.16%	patients with schizophrenia or schizoaffective disorder in first onset or acute exacerbation	7.47 (SD = 6.93)	NIP; data from three separate studies	NIP	NIP
	262	NIP on age mean	20.6%	Treatment group; patients meeting DSM-IV-TR criteria for diagnosis of schizophrenia	NIP on disease duration	NIP	USA	English
	130	NIP on age mean	15.49%	Placebo group; patients meeting DSM-IV-TR criteria for diagnosis of schizophrenia	NIP on disease duration	NIP	USA	English
Langeveld, J., et al. (2013). "Is there an optimal factor structure of the Positive and Negative Syndrome Scale in patients with first-episode psychosis?" Scand J Psychol 54(2): 160-165.	588	NIP on age mean	NIP on Gender	acutely psychotic patients meeting DSM-IV criteria for diagnosis of schizophrenia, schizophreniform disorder, schizoaffective disorder, brief psychotic episode, delusional disorder, affective psychosis with mood-incongruent psychotic features or psychotic disorder not otherwise specified	NIP on disease duration	In- and Outpatients	Norway& Denmark	Norwegian& Danish
Rodriguez-Jimenez, R., et al. (2013). "Cognition and the five-factor model of the positive and negative syndrome scale in schizophrenia." Schizophr Res 143(1): 77-83.	201	38.0 (SD = 9.7)	31.34%	stable patients meeting DSM-IV criteria for diagnosis of schizophrenia	NIP on disease duration	Outpatients, Hospital Universitario 12 de Octubre, Madrid and Hospital Virgen de la Luz, Cuenca	Spain	Spanish

		Population		Disease characteristics		Instrument administration		
**Ref**	**N**	**Age mean**	**Gender %female**	**Disease**	**Disease duration mean (SD) yr**	**Setting**	**Country**	**Language**
Higuchi, C. H., et al. (2014). "Factor structure of the Positive and Negative Syndrome Scale (PANSS) in Brazil: convergent validation of the Brazilian version." Braz J Psychiatry 36(4): 336-339.	292	33.64 (SD = 11.07)	34.6%	patients meeting DSM-IV criteria for diagnosis of schizophrenia	10.70 (SD = 9.67)	Universidade Federal de Sã o Paulo (UNIFESP), Brazil; Hospital Luzia de Pinho Melo; Santa Casa de Misericordia de Sa o Paulo, Brazil	Brazil	NIP (assumable Brazilian)
Khan, A., et al. (2014). "The evolution of illness phases in schizophrenia: A non-parametric item response analysis of the Positive and Negative Syndrome Scale." Schizophrenia Research: Cognition 1(2): 53-89.	305	23.6 (SD = 4.9)	27%	patients meeting DSM-IV criteria for diagnosis of schizophrenia or schizoaffective disorder; first episode	NIP on disease duration	Inpatients; New York State	USA	English
	694	NIP on Age	33,60%	patients meeting DSM-IV criteria for diagnosis of schizophrenia or schizoaffective disorder; Chronic Inpatients	NIP on disease duration	Inpatients; New York State	USA	English
	833	NIP on Age	38.9%	patients meeting DSM-IV criteria for diagnosis of schizophrenia or schizoaffective disorder; Chronic Ambulatory Outpatients	NIP on disease duration	Outpatients; New York State	USA	English
Stefanovics, E. A., et al. (2014). "A cross-national factor analytic comparison of three models of PANSS symptoms in schizophrenia." Psychiatry Res 219(2): 283-289.	1460	40.51 (SD = 11.09)	26%	patients meeting DSM-IV criteria for diagnosis of schizophrenia	NIP on disease duration	In- and Outpatients; international multicenter study	USA	English

		Population		Disease characteristics		Instrument administration		
Ref	N	Age mean	Gender %female	Disease	Disease duration mean (SD) yr	Setting	Country	Language
	252	35.47 (SD = 9.42)	33.7%	patients meeting DSM-IV criteria for diagnosis of schizophrenia	NIP on disease duration	Outpatients; Psychiatric Institute of the University of Sao Paulo	Brazil	assumable Brazilian
	498	50.03 (SD = 10.71)	32.7%	patients meeting DSM-IV criteria for diagnosis of schizophrenia	NIP on disease duration	Inpatients; Second Xiangya Hospital	China (Changsha)	Chinese
	522	49.41 (SD = 11.12)	33.7%	patients meeting DSM-IV criteria for diagnosis of schizophrenia	NIP on disease duration	Inpatients; Hui Long Guan Hospital in Beijing	China (Beijing)	Chinese
Van Erp, T. G. M., et al. (2014). "Converting positive and negative symptom scores between PANSS and SAPS/SANS." Schizophrenia Research 152(1): 289-294.	176	NIP on Age	NIP on Gender	patients meeting DSM-IV-R criteria for diagnosis of schizophrenia	NIP on disease duration	NIP; recruited from 1 of 7 sites	NIP (assumable USA)	NIP (assumable English)
Anderson, A., et al. (2015). "Sparse factors for the positive and negative syndrome scale: which symptoms and stage of illness?" Psychiatry Res 225(3): 283-290.	3647	NIP on Age	NIP on Gender	patients with schizophrenia	NIP on disease duration	NIP; information gathered from 11 different studies	36 countries	different languages
Xu, K., et al. (2015). "Preliminary analysis of positive and negative syndrome scale in ketamine-associated psychosis in comparison with schizophrenia." J Psychiatr Res 61: 64-72.	154	26.2 (SD = 9.4)	40%	inpatients on early course of schizophrenia	2.13 (SD = 0.075)	NIP	China	NIP (assumable Chinese)
	522	49.4 (SD = 11.1)	34%	inpatients of chronic schizophrenia	10.0 (SD = 9.5)	NIP	China	NIP (assumable Chinese)
Best, M. W., et al. (2016). "Examination of the Positive and Negative Syndrome Scale factor structure and longitudinal relationships with functioning in early psychosis." Early Interv Psychiatry 10(2): 165-170.	240	22.16 (SD = 5.83)	22.9%	inpatients participating in early intervention in psychosis program	NIP on disease duration	Inpatients; Kingston, Ontario	Canada	NIP (assumable English)

		Population		Disease characteristics		Instrument administration		
Ref	N	Age mean	Gender %female	Disease	Disease duration mean (SD) yr	Setting	Country	Language
Dragioti, E., et al. (2017). "Could PANSS be a useful tool in the determining of the stages of schizophrenia? A clinically operational approach." J Psychiatr Res 86: 66-72.	170	40.7 (SD = 11.6)	36.5%	patients meeting DSM-V criteria for diagnosis of schizophrenia	NIP on disease duration	Inpatients directly prior to discharge & outpatients	Greece	Greek
Hopkins, S. C., et al. (2017). "Understanding Antipsychotic Drug Treatment Effects: A Novel Method to Reduce Pseudospecificity of the Positive and Negative Syndrome Scale (PANSS) Factors." Innov Clin Neurosci 14(11-12): 54-58.	1710	NIP on Age	NIP on Gender	patients with acute exacerbation of schizophrenia	NIP on disease duration	NIP; information gathered from 5 different lurasidone treatment studies	NIP (assumable USA)	NIP (assumable English)
Yehya, A., et al. (2017). "Validation of the Five-Factor Model of the Arabic Version of the Positive and Negative Syndrome Scale in Schizophrenia." Psychopathology 50(3): 211-218.	101	35.03 (SD = 9.99)	33.7%	patients meeting DSM-IV-TR criteria for diagnosis of schizophrenia	11.69 (SD = 8.98)	NIP (assumably inpatients)	several arabic countries, predominantly Qatar	formal Arabic
Anderson, A. E., et al. (2018). "Bifactor Modeling of the Positive and Negative Syndrome Scale: Generalized Psychosis Spans Schizoaffective, Bipolar, and Schizophrenia Diagnoses." Schizophr Bull 44(6): 1204-1216.	5094	NIP on Age	NIP	patients with schizophrenia	NIP on disease duration	NIP; information gathered from 16 different studies	several countries	several languages
Hopkins, S. C., et al. (2018). "Transformed PANSS Factors Intended to Reduce Pseudospecificity Among Symptom Domains and Enhance Understanding of Symptom Change in Antipsychotic-Treated Patients With Schizophrenia." Schizophr Bull 44(3): 593-602.	1710	NIP on Age	NIP	patients with acute exacerbation of schizophrenia	NIP on disease duration	NIP; information gathered from 5 different lurasidone treatment studies	NIP (assumable USA)	NIP (assumable English)

		Population		Disease characteristics		Instrument administration		
**Ref**	**N**	**Age mean**	**Gender %female**	**Disease**	**Disease duration mean (SD) yr**	**Setting**	**Country**	**Language**
Østergaard, S. D., et al. (2016). "PANSS-6: a brief rating scale for the measurement of severity in schizophrenia." Acta Psychiatr Scand 133(6): 436-444.	229	38.3 (NO S	26%	NIP	NIP on dise	NIP	NIP	NIP
Lin, C. H., et al. (2018). "Early improvement in PANSS-30, PANSS-8, and PANSS-6 scores predicts ultimate response and remission during acute treatment of schizophrenia." Acta Psychiatr Scand 137(2): 98-108.	270	38.5 (SD = 8.5)	46.3 %	patients meeting DSM-IV criteria for diagnosis of schizophrenia	NIP on disease duration	inpatients; data from 3 trials; all conducted in Kai-Syuan Psychiatric Hospital, Kaohsiung, Tai- wan	Taiwan	NIP (assumable Taiwanese)
Lefort-Besnard, J., et al. (2018). "Patterns of schizophrenia symptoms: hidden structure in the PANSS questionnaire." Transl Psychiatry 8(1): 237.	218	NIP on age mean	29.36%	NIP	NIP on disease duration	NIP	International	several languages
Østergaard, S. D., et al. (2018). "The Validity and Sensitivity of PANSS-6 in the Clinical Antipsychotic Trials of Intervention Effectiveness (CATIE) Study." Schizophr Bull 44(2): 453-462.	1493	NIP on Age	NIP on Gender	patients meeting DSM-IV criteria for diagnosis of chronic but non-refractory schizophrenia	NIP on disease duration	NIP	USA	English
Østergaard, S. D., et al. (2018). "The validity and sensitivity of PANSS-6 in treatment-resistant schizophrenia." Acta Psychiatr Scand 138(5): 420-431.	56	NIP on Age	NIP on Gender	patients meeting DSM-IV criteria for diagnosis of treatment-resistant schizophrenia	NIP on disease duration	NIP	USA	English
Fountoulakis, K. N., et al. (2019). "Staging of Schizophrenia With the Use of PANSS: An International Multi-Center Study." Int J Neuropsychopharmacol 22(11): 681-697.	2358	37.21 (SD = 11.87) 39	39.40%	patients meeting DSM-IV/-V criteria for diagnosis of schizophrenia	NIP on disease duration	Inpatients	International multicenter study	NIP

		Population		Disease characteristics		Instrument administration		
**Ref**	**N**	**Age mean**	**Gender %female**	**Disease**	**Disease duration mean (SD) yr**	**Setting**	**Country**	**Language**
Freitas, R., et al. (2019). "Can the Positive and Negative Syndrome scale (PANSS) differentiate treatment-resistant from non-treatment-resistant schizophrenia? A factor analytic investigation based on data from the Pattern cohort study." Psychiatry Res 276: 210-217.	1429	42.2 (SD = 11.50)	29.46%	stable patients meeting the diagnostic criteria of schizophrenia according to the DSM-IV-TR or ICD 10	15.08 (SD = 10.41)	NIP	8 different countries	several languages
	1020	42.78 (SD = 11.88)	31.7%	non-treatment resistant stable patients meeting the diagnostic criteria of schizophrenia according to the DSM-IV-TR or ICD-10	14.60 (SD = 10.91)	NIP	8 different countries	several languages
	409	40.13 (SD = 10.25)	23.7%	treatment resistant stable patients meeting the diagnostic criteria of schizophrenia according to the DSM-IV-TR or ICD-10	16.27 (SD = 8.93)	NIP	8 different countries	several languages
Lim, K., et al. (2021). "Large-scale evaluation of the Positive and Negative Syndrome Scale (PANSS) symptom architecture in schizophrenia." Asian J Psychiatr 62: 102732.	3511	42.68 (SD = 12.35)	34.6%	patients meeting DSM-IV criteria for diagnosis of schizophrenia or classification of being UHR according CAARMS	16.63 (SD = 11.91)	NIP	USA, Singapur	English (possibly other languages)
Higuchi, C. H., et al. (2022). "Identifying strategies to improve PANSS based dimensional models in schizophrenia: Accounting for multilevel structure, Bayesian model and clinical staging." Schizophr Res 243: 424-430.	700	34.89 (SD = NIP)	35.7%	patients meeting DSM-IV criteria for diagnosis of schizophrenia	13.21 (SD = NIP)	NIP (assumably in-& outpatients)	Brazil, Sao Paulo	Portugese
Kagan, S., et al. (2022). "Longitudinal invariance of the positive and negative syndrome scale negative dimension in antipsychotic naïve first-episode schizophrenia." Early Interv Psychiatry 16(5): 581-586.	138	25.55 (SD = 7.158)	34.1%	antipsychotic naive first-episode patients meeting DSM-IV criteria for diagnosis of schizophrenia, schizoaffective or schizophreniform disorder	NIP on disease duration	Outpatients, Grupo de Atençao às Psicoses Iniciais(GAPI)	Brazil, Sao Paulo	Portugese

		Population		Disease characteristics		Instrument administration		
**Ref**	**N**	**Age mean**	**Gender %female**	**Disease**	**Disease duration mean (SD) yr**	**Setting**	**Country**	**Language**
Higuchi, C. H., et al. (2022). "Identifying strategies to improve PANSS based dimensional models in schizophrenia: Accounting for multilevel structure, Bayesian model and clinical staging." Schizophr Res 243: 424-430.	700	34.89 (SD = NIP)	35.7%	patients meeting DSM-IV criteria for diagnosis of schizophrenia	13.21 (SD = NIP)	NIP (assumably in-& outpatients)	Brazil, Sao Paulo	Portugese
Kagan, S., et al. (2022). "Longitudinal invariance of the positive and negative syndrome scale negative dimension in antipsychotic naïve first-episode schizophrenia." Early Interv Psychiatry 16(5): 581-586.	138	25.55 (SD = 7.158)	34.1%	antipsychotic naive first-episode patients meeting DSM-IV criteria for diagnosis of schizophrenia, schizoaffective or schizophreniform disorder	NIP on disease duration	Outpatients, Grupo de Atençao às Psicoses Iniciais(GAPI)	Brazil, Sao Paulo	Portugese
Kay, S. R. and S. Sevy (1990). "Pyramidical model of schizophrenia." Schizophr Bull 16(3): 537-545.	240	33.1 (SD = 10.21)	25.42%	patients meeting DSM-III criteria for diagnosis of schizophrenia	NIP on disease duration	Inpatients	USA, New York City	English
Lepine, J. (1991). "Dimensions positives et négatives dans les schizophrénies." Les Cahiers de prisme 1: 23-29.	331	34.8 (SD = 10.5)	36%	patients meeting DSM-III-R criteria for diagnosis of schizophrenia	NIP on disease duration	NIP	France, multicenter study	French
Lindström, E. and L. von Knorring (1993). "Principal component analysis of the Swedish version of the Positive and Negative Syndrome Scale for schizophrenia." Nordic Journal of Psychiatry 47(4): 257-263.	120	37.8 (SD = 10.5)	32.5% (female)	patients meeting DSM-III-R criteria for diagnosis of chronic schizophrenia	NIP on disease duration	NIP	Sweden	Swedish
Kawasaki, Y., et al. (1994). "Evaluation and interpretation of symptom structures in patients with schizophrenia." Acta Psychiatr Scand 89(6): 399-404.	70	26 (NIP on SD)	24.29%	patients meeting DSM-III-R criteria for diagnosis of schizophrenia or schizophreniform disorder	5 (NIP on SD)	In- and Outpatients; Department of Neuropsychia- try, Kanazawa University Hospital.	Japan	Japanese

		Population		Disease characteristics		Instrument administration		
Ref	N	Age mean	Gender %female	Disease	Disease duration mean (SD) yr	Setting	Country	Language
Higashima, M., et al. (1998). "P300 and the thought disorder factor extracted by factor-analytic procedures in schizophrenia." Biol Psychiatry 44(2): 115-120.	73	25.8 (NIP on SD)	24.66%	patients meeting DSM-III-R criteria for diagnosis of schizophrenia or schizoaffective disorders	NIP on disease duration	In- and Outpatients; Department of Neuropsychiatry, Kanazawa University Hospital	Japan	Japanese
Klapal, M., et al. (1998). "[The 5 factor model of childhood schizophrenia]." Nervenarzt 69(3): 238-242.	44	NIP on Age	56.82%	patients diagnosed with childhood-onnset schizophrenia	NIP on disease duration	In- and Outpatients	Germany	German
Lindenmayer, J. P., et al. (1994). "Five-factor model of schizophrenia. Initial validation." J Nerv Ment Dis 182(11): 631-638.	240	31 (NIP SD)	25.42%	patients meeting DSM-III criteria for schizophrenia	9 (NO SD)	Inpatients; selected hospital in New York City	USA, New York City	English
Lindenmayer, J. P., et al. (1994). "A new five factor model of schizophrenia." Psychiatr Q 65(4): 299-322.	517	37.9 (NIP SD)	NIP on Gender	Baseline: patients meeting DSM-III criteria for schizophrenia	NIP on disease duration	NIP	USA& Canada	English (assumable just English, possibly French as well)
	507	NIP on Age	NIP on Gender	Wash-Out: patients meeting DSM-III criteria for schizophrenia	NIP on disease duration	NIP	USA& Canada	English (assumable just English, possibly French as well)
Bunk, D., et al. (1999). "Symptom dimensions in the course of childhood-onset schizophrenia." Eur Child Adolesc Psychiatry 8 Suppl 1: I29-35.	44	13.0 (SD = 1.7)	62,50%	onset: patients meeting DSM-IV criteria for schizophrenia (25 cases) or schizophreniform, schizoaffective, and affective disorder (19 cases)	NIP on disease duration	NIP	Germany	German
	33	53.8 (SD = 8.9)	62,50%	follow-up: patients from above onset meeting DSM-IV criteria of schizophrenia	NIP on disease duration	NIP	Germany	German
Nakaya, M., et al. (1999). "Latent structures underlying schizophrenic symptoms: a five-dimensional model." Schizophr Res 39(1): 39-50.	100	31.8 (SD = 9.8)	56%	acutely exacerbated patients meeting DSM-III criteria for diagnosis of schizophrenia or schizoaffective disorder	7.01 (SD = 7.48)	Inpatients; Dokkyo University School of Medicine	Japan	Japanese

		Population		Disease characteristics		Instrument administration		
Ref	N	Age mean	Gender %female	Disease	Disease duration mean (SD) yr	Setting	Country	Language
Lykouras, L., et al. (2000). "Five-factor model of schizophrenic psychopathology: how valid is it?" Eur Arch Psychiatry Clin Neurosci 250(2): 93-100.	258	31.9 (SD = 11.1)	22.5%	patients meeting DSM-III-R criteria for diagnosis of schizophrenic disorders	9.5 (SD = 7.8)	Inpatients	Greece	Greek
Drake, R. J., et al. (2003). "The evolution of symptoms in the early course of non-affective psychosis." Schizophr Res 63(1-2): 171-179.	257	around 27 (NO SD)*	around 30%*	patients meeting DSM-IV criteria for schizophreniform disorder, schizophrenia, schizoaffective disorder, delusional disorder or psychosis not otherwise specified	NIP on disease duration	NIP	England	English
Honey, G. D., et al. (2003). "The functional neuroanatomy of schizophrenic subsyndromes." Psychol Med 33(6): 1007-1018.	100	35 (SD = 9.2)	23%	patients meeting DSM-IV criteria for diagnosis of schizophrenia	10.24 (SD = 10)	NIP;Bethlem Royal and Maudsley NHS Trust	England	English
Lee, K. H., et al. (2003). "The five symptom dimensions and depression in schizophrenia." Psychopathology 36(5): 226-233.	105	35 (SD = 9.3)	30.48%	patients meeting DSM-IV criteria for diagnosis of schizophrenia	13 (SD = 9.02)	In- and Outpatients	Australia	English
Lindenmayer, J. P., et al. (2004). "Effects of atypical antipsychotics on the syndromal profile in treatment-resistant schizophrenia." J Clin Psychiatry 65(4): 551-556.	157	40.8 (SD = 9.2)	15.3%	patients meeting DSM-III-R criteria for diagnosis of schizophrenia or schizoaffective disorder	19.5 (SD = 8.4)	Inpatients; psychiatric state hospitals in New York& South carolina	USA	English
Reichenberg, A., et al. (2005). "Stability in schizophrenia symptoms over time: findings from the Mount Sinai Pilgrim Psychiatric Center Longitudinal Study." J Abnorm Psychol 114(3): 363-372.	215	75.5 (SD = 9.2)	56.4%	patients meeting DM-III-R/-IV criteria for diagnosis of chronic schizophrenia	NIP on disease duration	Inpatient (at baseline); Pilgrim Psychiatric Center	USA	English
Kim, J. H., et al. (2012). "Evaluation of the factor structure of symptoms in patients with schizophrenia." Psychiatry Res 197(3): 285-289.	150	34.6 (SD = 10.0)	47.33%	patients meeting DSM-IV criteria for diagnosis of schizophrenia	8,7 (SD = 7.1)	Outpatients;	Republic of Korea	Korean

		Population		Disease characteristics		Instrument administration		
**Ref**	**N**	**Age mean**	**Gender %female**	**Disease**	**Disease duration mean (SD) yr**	**Setting**	**Country**	**Language**
Stochl, J., et al. (2014). "Multilevel ordinal factor analysis of the positive and negative syndrome scale (PANSS)." Int J Methods Psychiatr Res 23(1): 25-35.	507	42.2 (11.4)	33.7%	patients meeting ICD-10 criteria of schizophrenia or related psychotic disorder (F20–F29)	15.9 (SD = 10.3)	Outpatients; multicenter study (DIALOG study)	UK, Spain, Netherlands, Sweden, Germany, Switzerland	languages of the according countries
Levine, S. Z., et al. (2011). "Recommendations to improve the positive and negative syndrome scale (PANSS) based on item response theory." Psychiatry Res 188(3): 446-452	1882	NIP	29.4%	patients meeting DSM-III-R criteria for diagnosis of schizophrenia, schizophreniform disorder or schizoaffective disorder	NIP on disease duration	NIP	International (17 countries)	NIP
Anderson, A. E., et al. (2017). "Disparity between General Symptom Relief and Remission Criteria in the Positive and Negative Syndrome Scale (PANSS): A Post-treatment Bifactor Item Response Theory Model." Innov Clin Neurosci 14(11-12): 41-53.	3647	NIP on Age	38.77%	patients with schizophrenia	NIP on disease duration	NIP	NIP	NIP
Baandrup, L., et al. (2022). "Scalability of the Positive and Negative Syndrome Scale in first-episode schizophrenia assessed by Rasch models." Acta Psychiatrica Scandinavica 146(1): 21-35.	1073	NIP on Age	NIP on Gender	first-episode patients meeting DSM-IV or ICD-10 criteria of schizophrenia, schizophreniform disorder, or schizoaffective disorder	NIP on disease duration	NIP	3 European studies	NIP
Marder, S. R., et al. (1997). "The effects of risperidone on the five dimensions of schizophrenia derived by factor analysis: combined results of the North American trials." J Clin Psychiatry 58(12): 538-546.	513	37 (SD = 10.3)	17%	patients meeting DSM-III-R criteria for diagnosis of chronic schizophrenia	NIP on disease duration	NIP	North America	NIP (assumable English)
Findling, R. L., et al. (2023). "An Optimized Version of the Positive and Negative Symptoms Scale (PANSS) for Pediatric Trials." J Am Acad Child Adolesc Psychiatry 62(4): 427-434.	118	14.26 (SD = 2.41)	35%	youth patients with diagnosis of schizophrenia or schizoaffective disorder	NIP on disease duration	NIP	NIMH TEOSS study sample	NIP

		Population		Disease characteristics		Instrument administration		
Ref	N	Age mean	Gender %female	Disease	Disease duration mean (SD) yr	Setting	Country	Language
Cuesta, M. J. and V. Peralta (1995). "Psychopathological dimensions in schizophrenia." Schizophrenia bulletin 21(3): 473-482.	100	35.8 (SD=12.7)	31%	patients meeting DSM-III-R criteria for diagnosis of schizophrenia	10.4 (SD = 8.9)	Inpatients	Spain	Spanish
Dollfus, S. and B. Everitt (1998). "Symptom structure in schizophrenia: two-, three- or four-factor models?" Psychopathology 31(3): 120-130.	135	41.0 (SD =14.3)	50.4%	patients in currently either acute or stabilized illness phase of schizophrenia	NIP on disease duration	NIP	France	French
Mohr, P. E., et al. (2004). "The heterogeneity of schizophrenia in disease states." Schizophrenia research 71(1): 83-95.	663	38.3 (NO SD presented)	31.1%	patients with chronic schizophrenia	16.3 (No SD presented)	In- and Outpatients	USA	English
Rabinowitz, J., et al. (2006). "To what extent do the PANSS and CGI-S overlap?" Journal of clinical psychopharmacology 26(3): 303-307.	1362	NIP on Age	NIP on Gender	patients with chronic schizophrenia	NIP on disease duration	NIP	International multicenter study	
	519	NIP on Age	NIP on Gender	patients with acute schizophrenia	NIP on disease duration	NIP	USA	English
	533	NIP on Age	NIP on Gender	patients with schizophrenia, schizophreniform or schizoaffective disorder	NIP on disease duration	NIP	International multicenter study	several languages
	466	NIP on Age	NIP on Gender	patients with schizophrenia or schizoaffective disorder	NIP on disease duration	NIP	International multicenter study	several languages
	546	NIP on Age	NIP on Gender	symptomatically stable patients with schizophrenia	NIP on disease duration	NIP	International multicenter study	several languages
	283	NIP on Age	NIP on Gender	patients with acute schizophrenia	NIP on disease duration	NIP	USA	English
	582	NIP on Age	NIP on Gender	symptomatically stable patients with schizophrenia	NIP on disease duration	NIP	International multicenter study	several languages

		Population		Disease characteristics		Instrument administration		
**Ref**	**N**	**Age mean**	**Gender %female**	**Disease**	**Disease duration mean (SD) yr**	**Setting**	**Country**	**Language**
Villalta-Gil, V., et al. (2006). "Four symptom dimensions in outpatients with schizophrenia." Comprehensive psychiatry 47(5): 384-388.	219	39.6 (SD = 11.97)	35%	patients meeting DSM-IV criteria for diagnosis of schizophrenia	16.52 (SD = 10.13)	Outpatients; Sant Joan de De u–Mental Health Services Network, Barcelona area	Spain	Spanish
Ruhrmann, S., et al. (2007). "Efficacy of flupentixol and risperidone in chronic schizophrenia with predominantly negative symptoms." Progress in neuro-psychopharmacology & biological psychiatry 31(5): 1012-1022.	144	40.39 (SD = 11.98)	37.5%	patients meeting ICD-10 criteria for diagnosis of schizophrenia	11.39 (SD = 9.99)	In- and Outpatients; Multicenter	Austria& Germany	German
Tirupati, S. N., et al. (2006). "Psychopathology in never-treated schizophrenia." Comprehensive psychiatry 47(1): 1-6.	143	47 (SD = 16.3)	50.35%	patients with never-treated schizophrenia	10.7 (SD = 9.1)	NIP	India	Indian
Ruiz-Veguilla, M., et al. (2008). "Neurodevelopmental markers in different psychopathological dimensions of first episode psychosis: the ESPIGAS study." European psychiatry : the journal of the Association of European Psychiatrists 23(8): 533-540.	87	27 (SD = 10)	37%	patients with psychosis (DSM-IV 295-298)	NIP on disease duration	In- and Outpatients	Spain	Spanish
Thokagevistk, K., et al. (2016). "Validation of disease states in schizophrenia: comparison of cluster analysis between US and European populations." Journal of market access & health policy 4.	1208	NIP on Age	NIP on Gender	patients meeting DSM-IV criteria for diagnosis of schizophrenia	NIP on disease duration	NIP	France, Germany, Britain	French, German, English
Grover, S., et al. (2018). "Factor analysis of symptom dimensions (psychotic, affective and obsessive compulsive symptoms) in schizophrenia." Asian journal of psychiatry 38: 72-77.	181	34 (SD = 11.91)	46.4%	patients meeting DSM-IV criteria for diagnosis of schizophrenia	10.78 (SD = 8,63)	In- and Outpatients; tertiary care hospital in North India	India	NIP (assumable Indian)

		Population		Disease characteristics		Instrument administration		
Ref	N	Age mean	Gender %female	Disease	Disease duration mean (SD) yr	Setting	Country	Language
Petruzzelli, M. G., et al. (2018). "Early onset first episode psychosis: dimensional structure of symptoms, clinical subtypes and related neurodevelopmental markers." European child & adolescent psychiatry 27(2): 171-179.	60	14 (SD = 2.88)	38.3%	patients meeting DSM-IV criteria for diagnosis of early onset first episode schizophrenia spectrum psychosis (schizophrenia, schizophreniphorm disorder, schizoaffective disorder, psychosis not otherwise specified)	0.27 (SD = 0.15)	Inpatients; Child Neuropsychiatry Unit, Department of Basic Medical Sciences, Neurosciences and Sense Organs, University of Bari "Aldo Moro"	Italy	Italian
Golay, P., et al. (2022). "Symptom dimensions stability over time in recent onset psychosis: A prospective study." Schizophrenia research 246: 126-131.	362	24.75 (SD = 4.79)	33.3%	patients meeting the criteria for psychosis as defined by the 'psychosis threshold' subscale in the Comprehensive Assessment of At-Risk Mental States (CAARMS)	NIP on disease duration	NIP; Lausanne University Hospital's Department of Psychiatry	Switzerland	NIP (assumable French)
Walsh-Messinger, J., et al. (2018). "Factor Structure of the Positive and Negative Syndrome Scale (PANSS) Differs by Sex." Clin Schizophr Relat Psychoses 11(4): 207-213.	123	31.85 (SD = 10.79)	0%	patients meeting DSM-IV criteria for diagnosis of schizophrenia or schizoaffective disorder	NIP on disease duration	Inpatients	USA	English
	74	33.24 (SD = 8.88)	100%	patients meeting DSM-IV criteria for diagnosis of schizophrenia or schizoaffective disorder	NIP on disease duration	Inpatients	USA	English
Peitl, V., et al. (2017). "Depressive symptoms in schizophrenia and dopamine and serotonin gene polymorphisms." Prog Neuropsychopharmacol Biol Psychiatry 77: 209-215.	300	39.60 (SD = 14.07)	27.3%	patients meeting DSM-IV-TR criteria for diagnosis of schizophrenia	1.86 (SD = 0.89)	Inpatients; Sestre Milosrdnice University Hospital Center	Croatia	Croatian

		Population		Disease characteristics		Instrument administration		
**Ref**	**N**	**Age mean**	**Gender %female**	**Disease**	**Disease duration mean (SD) yr**	**Setting**	**Country**	**Language**
Fong, T. C. T., et al. (2015). "Psychometric validation of the consensus five-factor model of the Positive and Negative Syndrome Scale." Comprehensive Psychiatry 62: 204-208.	146	53.9 (SD = 8.4)	46.9%	patients meeting DSM-IV-TR criteria for diagnosis of schizophrenia	NIP on disease duration	NIP; mental health rehab hostel in Hong Kong	China	Chinese
Woodward, T. S., et al. (2014). "Symptom changes in five dimensions of the Positive and Negative Syndrome Scale in refractory psychosis." Eur Arch Psychiatry Clin Neurosci 264(8): 673-682.	610	36.68 (SD = 11.34)	39%	patients meeting DSM-III-R or DSM-IV criteria for disease with refractory psychosis (schizophrenia, schizoaffecive disorder, bipolar disorder, major depression)	NIP on disease duration	Inpatients; Treatment Refractory Psychosis Program at Riverview Hospital, Port Coquitlam, British Columbia	Canada	English
Hayashi, N., et al. (2002). "Is there a gender difference in a factorial structure of the positive and negative syndrome scale? A test by structural equation modeling." Psychopathology 35(1): 28-35.	154	46.8 (SD = 12.0)	0%	patients meeting DSM-IV criteria for diagnosis of schizophrenia	22.3 (SD = 12.2)	In- and Outpatients; Tokyo Metropolitan Matsuzawa Hospital, Tokyo	Japan	Japanese
	154	48.1 (SD = 13.0)	100%	patients meeting DSM-IV criteria for diagnosis of schizophrenia	19.6 (SD = 11.8)	In- and Outpatients; Tokyo Metropolitan Matsuzawa Hospital, Tokyo	Japan	Japanese
*no precise information provided by the authors; NIP = no information provided								

Appendix 5 - Correlations for construct validity and respective hypotheses

M5M-factor	reference	correlated scales	correlation coefficient	total patient number	meta-analysis/single study	number of single correlations entering meta-analysis	hypothesis applied*	hypothesis fulfilled?
**Marder Anxiety Depression Factor**	Rabinowitz et al. 2006	Marder anxiety depression factor <-> CGI-S	0.182	4291	MA	7	2	yes
	Hopkins et al. 2017	Marder anxiety/depression factor <-> Marder total score	0.640	1710	SS	/	2	no
**Marder Disorganized Factor**	Rabinowitz et al. 2006	Marder disorganized factor <-> CGI-S	0.520	4291	MA	7	2	yes
	Hopkins et al. 2017	Marder disorganized factor <-> Marder depression/anxiety factor	0.340	1710	SS	/	2	yes
	Hopkins et al. 2017	Marder disorganized factor <-> Marder hostility/excitement factor	0.560	1710	SS	/	2	yes
	Hopkins et al. 2017	Marder disorganized thinking factor <-> Marder total score	0.840	1710	SS	/	2	no
**Marder Hostility/ Excitement Facto**	Rabinowitz et al. 2006	Marder hostility factor <-> CGI-S	0.345	4291	MA	7	2	yes
	Hopkins et al. 2017	Marder hostility/excitement factor <-> Marder depression/anxiety factor	0.470	1710	SS	/	3	no
	Hopkins et al. 2017	Marder hostility/excitement factor <-> Marder total score	0.760	1710	SS	/	2	no
**Marder Negative Factor**	Rabinowitz et al. 2006	Marder negative factor <-> CGI-S	0.424	4291	MA	7	2	yes
	Hopkins et al. 2017	Marder negative factor <-> Marder anxiety/depression factor	0.370	1710	SS	/	2	yes
	Hopkins et al. 2017	Marder negative factor <-> Marder disorganized thinking factor	0.610	1710	SS	/	2	no
	Hopkins et al. 2017	Marder negative factor <-> Marder hostility/excitement factor	0.350	1710	SS	/	3	yes
	Hopkins et al. 2017	Marder negative factor <-> Marder total score	0.740	1710	SS	/	2	no
	Van Erp et al. 2014	Marder negative factor <-> SANS	0.831	352	MA	2	1	yes
	Van Erp et al. 2014	Marder negative factor <-> SAPS	0.265	352	MA	2	3	yes
**Marder Positive Factor**	Rabinowitz et al. 2006	Marder positive factor <-> CGI-S	0.555	4291	MA	7	2	yes
	Hopkins et al. 2017	Marder positive factor <-> Marder depression/anxiety factor	0.520	1710	SS	/	2	yes
	Hopkins et al. 2017	Marder positive factor <-> Marder disorganized thinking factor	0.680	1710	SS	/	2	no
	Hopkins et al. 2017	Marder positive factor <-> Marder hostility/excitement factor	0.650	1710	SS	/	2	no
	Hopkins et al. 2017	Marder positive factor <-> Marder negative factor	0.409	1886	MA	2	3	no
	Hopkins et al. 2017	Marder positive factor <-> Marder total score	0.890	1710	SS	/	2	no
	Van Erp et al. 2014	Marder positive factor <-> SANS	0.310	352	MA	2	3	yes
	Van Erp et al. 2014	Marder positive factor <-> SAPS	0.736	352	MA	2	1	yes

*hypotheses: (1) Between instruments measuring the same or a very similar construct, we expected a correlation of ≥0.5. (2) Between instruments measuring related but different constructs, we expected a correlation of ≤0.6. (3) Between instruments measuring dissimilar or contrary constructs, we expected a correlation of ≤0.4.

Appendix 6 - Results from meta analysis for convergent validity

Standard random-effects meta-analyses were conducted using Comprehensive Meta-Analysis Version 2 and pooled correlation coefficients calculated. The rows without a study name contain the meta-analytically pooled results from single studies in the respective blocks above them.

Study name	Comparison	Correlation	Lower limit	Upper limit	Z-Value	p-Value	Total
Rabinowitz2006a	MarderAnxietyDepressionFactorCGIS	0,080	0,027	0,133	2,955	0,003	1362
Rabinowitz2006b	MarderAnxietyDepressionFactorCGIS	0,120	0,034	0,204	2,739	0,006	519
Rabinowitz2006c	MarderAnxietyDepressionFactorCGIS	0,270	0,189	0,347	6,374	0,000	533
Rabinowitz2006d	MarderAnxietyDepressionFactorCGIS	0,130	0,040	0,218	2,813	0,005	466
Rabinowitz2006e	MarderAnxietyDepressionFactorCGIS	0,270	0,190	0,346	6,452	0,000	546
Rabinowitz2006f	MarderAnxietyDepressionFactorCGIS	0,050	-0,067	0,166	0,837	0,402	283
Rabinowitz2006g	MarderAnxietyDepressionFactorCGIS	0,330	0,256	0,401	8,249	0,000	582
		0,182	0,097	0,264	4,183	0,000	4291
Hopkins2017	MarderAnxietyDepressionFactorMarderTotalScore	0,640	0,611	0,667	31,325	0,000	1710
		0,640	0,611	0,667	31,325	0,000	1710
Rabinowitz2006a	MarderDisorganizedFactorCGIS	0,430	0,386	0,472	16,954	0,000	1362
Rabinowitz2006b	MarderDisorganizedFactorCGIS	0,640	0,586	0,688	17,222	0,000	519
Rabinowitz2006c	MarderDisorganizedFactorCGIS	0,530	0,466	0,588	13,586	0,000	533
Rabinowitz2006d	MarderDisorganizedFactorCGIS	0,480	0,407	0,547	11,253	0,000	466
Rabinowitz2006e	MarderDisorganizedFactorCGIS	0,550	0,489	0,606	14,410	0,000	546
Rabinowitz2006f	MarderDisorganizedFactorCGIS	0,360	0,254	0,457	6,307	0,000	283
Rabinowitz2006g	MarderDisorganizedFactorCGIS	0,600	0,545	0,650	16,679	0,000	582
		0,520	0,447	0,585	11,909	0,000	4291
Hopkins2017	MarderDisorganizedFactorMarderAnxietyDepressionFactor	0,340	0,297	0,381	14,630	0,000	1710
		0,340	0,297	0,381	14,630	0,000	1710
Hopkins2017	MarderDisorganizedFactorMarderHostilityExcitementFactor	0,560	0,527	0,592	26,146	0,000	1710
		0,560	0,527	0,592	26,146	0,000	1710
Hopkins2017	MarderDisorganizedFactorMarderTotalScore	0,840	0,825	0,853	50,454	0,000	1710
		0,840	0,825	0,853	50,454	0,000	1710
Rabinowitz2006a	MarderHostilityExcitementFactorCGIS	0,290	0,241	0,338	11,007	0,000	1362
Rabinowitz2006b	MarderHostilityExcitementFactorCGIS	0,470	0,400	0,534	11,587	0,000	519
Rabinowitz2006c	MarderHostilityExcitementFactorCGIS	0,490	0,423	0,552	12,341	0,000	533
Rabinowitz2006d	MarderHostilityExcitementFactorCGIS	0,350	0,268	0,427	7,863	0,000	466
Rabinowitz2006e	MarderHostilityExcitementFactorCGIS	0,230	0,149	0,308	5,457	0,000	546
Rabinowitz2006f	MarderHostilityExcitementFactorCGIS	0,230	0,117	0,338	3,919	0,000	283
Rabinowitz2006g	MarderHostilityExcitementFactorCGIS	0,320	0,245	0,391	7,980	0,000	582
		0,345	0,267	0,418	8,181	0,000	4291
Hopkins2017	MarderHostilityExcitementFactorMarderAnxietyDepressionFactor	0,470	0,432	0,506	21,074	0,000	1710
		0,470	0,432	0,506	21,074	0,000	1710
Hopkins2017	MarderHostilityExcitementFactorMarderTotalScore	0,760	0,739	0,779	41,159	0,000	1710
		0,760	0,739	0,779	41,159	0,000	1710
Rabinowitz2006a	MarderNegativeFactorCGIS	0,310	0,261	0,357	11,817	0,000	1362
Rabinowitz2006b	MarderNegativeFactorCGIS	0,330	0,251	0,405	7,788	0,000	519
Rabinowitz2006c	MarderNegativeFactorCGIS	0,670	0,620	0,714	18,665	0,000	533
Rabinowitz2006d	MarderNegativeFactorCGIS	0,360	0,278	0,437	8,110	0,000	466
Rabinowitz2006e	MarderNegativeFactorCGIS	0,500	0,434	0,560	12,800	0,000	546
Rabinowitz2006f	MarderNegativeFactorCGIS	0,180	0,065	0,291	3,045	0,002	283
Rabinowitz2006g	MarderNegativeFactorCGIS	0,520	0,458	0,577	13,868	0,000	582
		0,424	0,297	0,536	6,076	0,000	4291
Hopkins2017	MarderNegativeFactorMarderAnxietyDepressionFactor	0,370	0,328	0,410	16,048	0,000	1710
		0,370	0,328	0,410	16,048	0,000	1710
Hopkins2017	MarderNegativeFactorMarderDisorganizedFactor	0,610	0,579	0,639	29,290	0,000	1710
		0,610	0,579	0,639	29,290	0,000	1710
Hopkins2017	MarderNegativeFactorMarderHostilityExcitementFactor	0,350	0,308	0,391	15,099	0,000	1710
		0,350	0,308	0,391	15,099	0,000	1710
Hopkins2017	MarderNegativeFactorMarderTotalScore	0,740	0,718	0,761	39,270	0,000	1710
		0,740	0,718	0,761	39,270	0,000	1710
VanErp2014a	MarderNegativeFactorSANS	0,850	0,803	0,886	16,522	0,000	176
VanErp2014b	MarderNegativeFactorSANS	0,810	0,752	0,855	14,824	0,000	176
		0,831	0,788	0,866	18,457	0,000	352
VanErp2014a	MarderNegativeFactorSAPS	0,250	0,106	0,384	3,359	0,001	176
VanErp2014b	MarderNegativeFactorSAPS	0,280	0,138	0,411	3,784	0,000	176
		0,265	0,165	0,360	5,051	0,000	352
Rabinowitz2006a	MarderPositiveFactorCGIS	0,460	0,417	0,501	18,333	0,000	1362
Rabinowitz2006b	MarderPositiveFactorCGIS	0,570	0,509	0,625	14,709	0,000	519
Rabinowitz2006c	MarderPositiveFactorCGIS	0,580	0,521	0,634	15,251	0,000	533
Rabinowitz2006d	MarderPositiveFactorCGIS	0,570	0,505	0,628	13,933	0,000	466
Rabinowitz2006e	MarderPositiveFactorCGIS	0,550	0,489	0,606	14,410	0,000	546
Rabinowitz2006f	MarderPositiveFactorCGIS	0,500	0,407	0,583	9,192	0,000	283
Rabinowitz2006g	MarderPositiveFactorCGIS	0,640	0,589	0,686	18,243	0,000	582
		0,555	0,502	0,604	16,636	0,000	4291
Hopkins2017	MarderPositiveFactorMarderAnxietyDepressionFactor	0,520	0,485	0,554	23,812	0,000	1710
		0,520	0,485	0,554	23,812	0,000	1710
Hopkins2017	MarderPositiveFactorMarderDisorganizedFactor	0,680	0,654	0,705	34,256	0,000	1710
		0,680	0,654	0,705	34,256	0,000	1710
Hopkins2017	MarderPositiveFactorMarderHostilityExcitementFactor	0,650	0,622	0,677	32,032	0,000	1710
		0,650	0,622	0,677	32,032	0,000	1710
Hopkins2017	MarderPositiveFactorMarderNegativeFactor	0,500	0,464	0,535	22,695	0,000	1710
VanErp2014	MarderPositiveFactorMarderNegativeFactor	0,290	0,148	0,420	3,927	0,000	176
		0,409	0,187	0,591	3,476	0,001	1886
Hopkins2017	MarderPositiveFactorMarderTotalScore	0,890	0,880	0,899	58,748	0,000	1710
		0,890	0,880	0,899	58,748	0,000	1710
VanErp2014a	MarderPositiveFactorSANS	0,290	0,148	0,420	3,927	0,000	176
VanErp2014b	MarderPositiveFactorSANS	0,330	0,191	0,456	4,509	0,000	176
		0,310	0,212	0,402	5,965	0,000	352
VanErp2014a	MarderPositiveFactorSAPS	0,710	0,628	0,776	11,669	0,000	176
VanErp2014b	MarderPositiveFactorSAPS	0,760	0,690	0,816	13,103	0,000	176
		0,736	0,683	0,781	17,274	0,000	352

Correlation and 95% CI

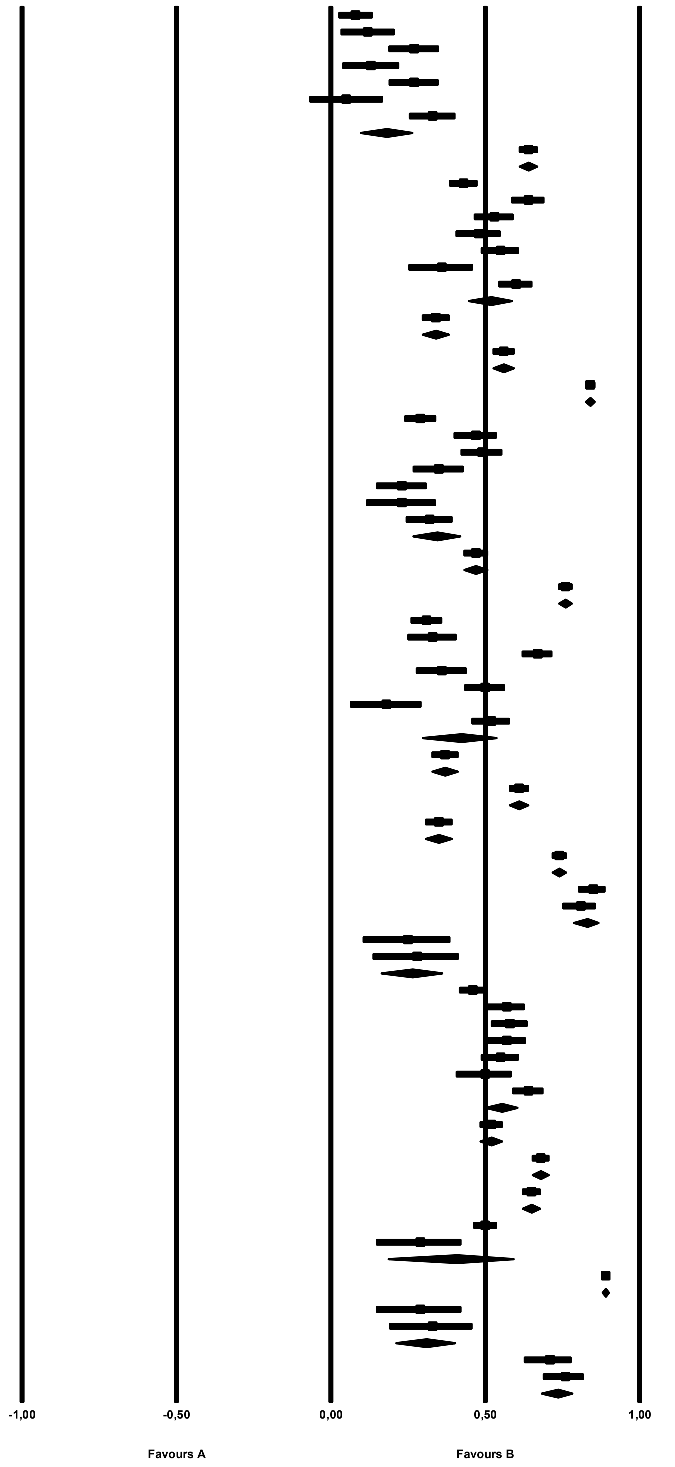

Appendix7 - Information on feasibility

**Feasibility aspects**	**PANSS (Positive and Negative Syndrome Scale)**
patient´s comprehensibility	not applicable (due to the PANSS being a ClinROM)
clinician´s comprehensibility	application of the PANSS might be challenging for inexpierenced users; rater trainings are required;
Type and ease of administrations	clinical interview, see 'Completion time' and 'Availability in different setting' below
Length of the instrument	30 items
Completion time	30-50 mins (according to Kay et al. 1987)
Patient's required mental and physical ability level	no specific level required
Ease of standardization	Structured clinical interview (SCI-PANSS) for PANSS simplifies standardization; rater trainings (e.g. provided by PANSS institute) make the standardization laborious
Ease of score calculation	score calculation by adding up item/subscale ratings; 30 points must always be deducted from PANSS scores when calculating percentage changes
Copyright	PANSS institute as copyright holder, usage of the scale needs licensing
Cost of an instrument	PANSS Technical Manual ($96,40), 25 PANSS Rating and Profile Forms ($90,80)
Required equipment	none except for rating forms
Availability in different settings	analog form, good in research setting, over 40 official language versions, clinical use problematic due to the instruments lenght
Regulatory agency's requirement for approval	none for the assessment of the PANSS, PANSS ratings necessary for EMA drug approval

Appendix8 - Confirmatory analyses of further PANSS models

CFAs of other models than Marder or the Three-factor Kay model					
reference	model	Factors	CFI	RMSEA	*Updated criteria* rating[a]
Hayashi, N., et al. (2002). "Is there a gender difference in a factorial structure of the positive and negative syndrome scale? A test by structural equation modeling." Psychopathology 35(1): 28-35.	5-factor model	5	0.901	/	(-)
Fitzgerald, P. B., et al. (2003). "A confirmatory factor analytic evaluation of the pentagonal PANSS model." Schizophr Res 61(1): 97-104.	"pentagonal model"	5	0.789	/	(-)
	"alternative five factor model"	5	0.719	/	(-)
Van Den Oord, E. J. C. G., et al. (2006). "Factor structure and external validity of the PANSS revisited." Schizophrenia Research 82(2-3): 213-223.	4-factor model	4	0.856	/	(-)
	5-factor model	5	0.909	/	(-)
	6-factor model	6	0.944	/	(-)
van der Gaag, M., et al. (2006). "The five-factor model of the Positive and Negative Syndrome Scale I: confirmatory factor analysis fails to confirm 25 published five-factor solutions." Schizophr Res 85(1-3): 273-279.	White, 1997	5	0.732	/	(-)
	Lindstrom, 1993	5	0.727	/	(-)
	Peuskens, 1992	5	0.724	/	(-)
	Lykouras, 2000	5	0.719	/	(-)
	Lancon, 1998a	5	0.715	/	(-)
	Lindenmayer, 1995a	5	0.709	/	(-)
	Lancon, 2000	5	0.708	/	(-)
	Fredrikson, 1997	5	0.706	/	(-)
	Wolthaus, 2000	5	0.704	/	(-)
	Emsley, 2003	5	0.702	/	(-)
	Mass, 2000	5	0.697	/	(-)
	Lindenmayer, 1994	5	0.683	/	(-)
	Lee, 2003	5	0.674	/	(-)
	Higashima, 1998a	5	0.671	/	(-)
	Bell, 1994 (WHVA)	5	0.651	/	(-)
	Bell, 1994 (Bronx)	5	0.648	/	(-)
	White, 1994a	5	0.639	/	(-)
	Kawasaki, 1994a	5	0.621	/	(-)
	Dollfus, 1995 (2)	5	0.588	/	(-)
	Dollfus, 1995 (1)	5	0.579	/	(-)
	Dollfus, 1991	5	0.543	/	(-)
	Lancon, 1999	5	0.539	/	(-)
van der Gaag, M., et al. (2006). "The five-factor model of the Positive and Negative Syndrome Scale II: a ten-fold cross-validation of a revised model." Schizophr Res 85(1-3): 280-287	5-factor model resulting from 10-fold cross-validation	5	0.905	/	(-)
Wallwork, R. S., et al. (2012). "Searching for a consensus five-factor model of the Positive and Negative Syndrome Scale for schizophrenia." Schizophr Res 137(1-3): 246-250.	Wallwork 5-factor model	5	0.950	/	(-)
	Wallwork 5-factor model	5	0.941	/	(-)
Jiang, J., et al. (2013). "Validated five-factor model of positive and negative syndrome scale for schizophrenia in Chinese population." Schizophr Res 143(1): 38-43.	Mass et al. (2000)	5	0.907	/	(-)
	van der Gaag et al. (2006a,b)	5	0.903	/	(-)
	White et al. (1997)	5	0.926	/	(-)
	Lindenmayer et al. (1994)	5	0.830	/	(-)
	Lindenmayer et al. (1995a)	5	0.884	/	(-)
	Dollfus and Petit (1995)	5	0.824	/	(-)

CFAs of other models than Marder or the Three-factor Kay model					
reference	model	Factors	CFI	RMSEA	*Updated criteria* rating[a]
	Lancon et al. (1998)	5	0.897	/	(-)
	Lancon et al. (2000) (acute)	5	0.899	/	(-)
	Lancon et al. (2000) (chronic)	5	0.900	/	(-)
	Bell et al. (1994) (Bronx)	5	0.870	/	(-)
	Bell et al. (1994) (WHVA)	5	0.835	/	(-)
	Kawasaki et al. (1994)	5	0.768	/	(-)
	Lindström and Von Knorring (1993)	5	0.893	/	(-)
	Citrome et al. (2011)	5	0.870	/	(-)
	Drake et al. (2003)	5	0.772	/	(-)
	Emsley et al. (2003)	5	0.857	/	(-)
	Kay and Sevy (1990)	4	0.824	/	(-)
	Lykouras et al. (2000)	5	0.887	/	(-)
	Lee et al. (2003)	5	0.829	/	(-)
	Higashima et al. (1998)	5	0.860	/	(-)
	Bunk et al. (1999)	5	0.794	/	(-)
	Wolthaus et al. (2000)	5	0.844	/	(-)
	Wallwork et al. (2012)	5	0.918	/	(-)
	Van den Oord et al. (2006)	5	0.728	/	(-)
	Kim et al. (2012)	5	0.865	/	(-)
	Fredrikson et al. (1997)	5	0.882	/	(-)
	Levine and Rabinowitz (2007)	5	0.856	/	(-)
	Fresan et al. (2005)	5	0.804	/	(-)
	Fitzgerald et al. (2003)	5	0.907	/	(-)
	Lindenmayer et al. (1995b)	5	0.888	/	(-)
	El Yazaji et al. (2002)	5	0.857	/	(-)
	Derived five-factor model	5	0.980	/	**(+)**
	Mass et al. (2000)	5	0.815	/	(-)
	van der Gaag et al. (2006a,b)	5	0.853	/	(-)
	White et al. (1997)	5	0.887	/	(-)
	Lindenmayer et al. (1994)	5	0.827	/	(-)
	Lindenmayer et al. (1995a)	5	0.842	/	(-)
	Dollfus and Petit (1995)	5	0.808	/	(-)
	Lancon et al. (1998)	5	0.830	/	(-)
	Lancon et al. (2000) (acute)	5	0.846	/	(-)
	Lancon et al. (2000) (chronic)	5	0.845	/	(-)
	Bell et al. (1994) (Bronx)	5	0.847	/	(-)
	Bell et al. (1994) (WHVA)	5	0.832	/	(-)
	Kawasaki et al. (1994)	5	0.779	/	(-)
	Lindström and Von Knorring (1993)	5	0.836	/	(-)
	Citrome et al. (2011)	5	0.838	/	(-)
	Drake et al. (2003)	5	0.791	/	(-)
	Emsley et al. (2003)	5	0.833	/	(-)
	Kay and Sevy (1990)	4	0.817	/	(-)

CFAs of other models than Marder or the Three-factor Kay model

reference	model	Factors	CFI	RMSEA	*Updated criteria* rating[a]
	Lykouras et al. (2000)	5	0.856	/	(-)
	Lee et al. (2003)	5	0.808	/	(-)
	Higashima et al. (1998)	5	0.838	/	(-)
	Bunk et al. (1999)	5	0.780	/	(-)
	Wolthaus et al. (2000)	5	0.828	/	(-)
	Wallwork et al. (2012)	5	0.854	/	(-)
	Van den Oord et al. (2006)	5	0.858	/	(-)
	Kim et al. (2012)	5	0.852	/	(-)
	Fredrikson et al. (1997)	5	0.824	/	(-)
	Levine and Rabinowitz (2007)	5	0.823	/	(-)
	Fresan et al. (2005)	5	0.807	/	(-)
	Fitzgerald et al. (2003)	5	0.862	/	(-)
	Lindenmayer et al. (1995b)	5	0.824	/	(-)
	El Yazaji et al. (2002)	5	0.829	/	(-)
	Derived five-factor model	5	0.915	/	(-)
	Mass et al. (2000)	5	0.882	/	(-)
	van der Gaag et al. (2006a,b)	5	0.900	/	(-)
	White et al. (1997)	5	0.902	/	(-)
	Lindenmayer et al. (1994)	5	0.866	/	(-)
	Lindenmayer et al. (1995a)	5	0.896	/	(-)
	Dollfus and Petit (1995)	5	0.839	/	(-)
	Lancon et al. (1998)	5	0.885	/	(-)
	Lancon et al. (2000) (acute)	5	0.884	/	(-)
	Lancon et al. (2000) (chronic)	5	0.896	/	(-)
	Bell et al. (1994) (Bronx)	5	0.891	/	(-)
	Bell et al. (1994) (WHVA)	5	0.855	/	(-)
	Kawasaki et al. (1994)	5	0.801	/	(-)
	Lindström and Von Knorring (1993)	5	0.887	/	(-)
	Citrome et al. (2011)	5	0.884	/	(-)
	Drake et al. (2003)	5	0.801	/	(-)
	Emsley et al. (2003)	5	0.879	/	(-)
	Kay and Sevy (1990)	4	0.840	/	(-)
	Lykouras et al. (2000)	5	0.891	/	(-)
	Lee et al. (2003)	5	0.858	/	(-)
	Higashima et al. (1998)	5	0.863	/	(-)
	Bunk et al. (1999)	5	0.801	/	(-)
	Wolthaus et al. (2000)	5	0.865	/	(-)
	Wallwork et al. (2012)	5	0.891	/	(-)
	Van den Oord et al. (2006)	5	0.848	/	(-)
	Kim et al. (2012)	5	0.878	/	(-)
	Fredrikson et al. (1997)	5	0.873	/	(-)
	Levine and Rabinowitz (2007)	5	0.877	/	(-)
	Fresan et al. (2005)	5	0.829	/	(-)

CFAs of other models than Marder or the Three-factor Kay model					
reference	model	Factors	CFI	RMSEA	*Updated criteria* rating[a]
	Fitzgerald et al. (2003)	5	0.884	/	(-)
	Lindenmayer et al. (1995b)	5	0.881	/	(-)
	El Yazaji et al. (2002)	5	0.863	/	(-)
	Derived five-factor model	5	0.948	/	(-)
Langeveld, J., et al. (2013). "Is there an optimal factor structure of the Positive and Negative Syndrome Scale in patients with first-episode psychosis?" Scand J Psychol 54(2): 160-165.	five factor model Emsley	5	0.726	/	(-)
	five factor model White	5	0.782	/	(-)
	five factor model Van der Gaag	5	0.761	/	(-)
	five factor model Wallwork/ Fortgang	5	0.812	/	(-)
Rodriguez-Jimenez, R., et al. (2013). "Cognition and the five-factor model of the positive and negative syndrome scale in schizophrenia." Schizophr Res 143(1): 77-83.	Wallwork 5-factor model	5	0.93	/	(-)
Stefanovics, E. A., et al. (2014). "A cross-national factor analytic comparison of three models of PANSS symptoms in schizophrenia." Psychiatry Res 219(2): 283-289.	Wallwork	5	/	0.0842	(-)
	van der Gaag	5	/	0.0817	(-)
	Wallwork	5	/	0.084	(-)
	van der Gaag	5	/	0.0924	(-)
	Wallwork	5	/	0.0828	(-)
	van der Gaag	5	/	0.0914	(-)
	Wallwork	5	/	0.0978	(-)
	van der Gaag	5	/	0.0939	(-)
Anderson, A., et al. (2015). "Sparse factors for the positive and negative syndrome scale: which symptoms and stage of illness?" Psychiatry Res 225(3): 283-290.	post-treatment 18 items retained	5	0.761	/	(-)
	pre-treatment 18 items retained	5	0.825	/	(-)
	post-treatment 20 items retained	5	0.712	/	(-)
	pre-treatment 20 items retained	5	0.811	/	(-)
	post-treatment proposed model	5	0.689	/	(-)
	pre-treatment proposed model	5	0.802	/	(-)
Anderson, A. E., et al. (2018). "Bifactor Modeling of the Positive and Negative Syndrome Scale: Generalized Psychosis Spans Schizoaffective, Bipolar, and Schizophrenia Diagnoses." Schizophr Bull 44(6): 1204-1216.	derived bifactorial model	Bifactorial[b]	/	0.09	(-)
	Bifactorial model prespecified from previous research	Bifactorial[b]	/	0.09	(-)
	5-factor model, not further specified	5	/	0.11	(-)
	6-factor model, not further specified	6	/	0.10	(-)
Freitas, R., et al. (2019). "Can the Positive and Negative Syndrome scale (PANSS) differentiate treatment-resistant from non-treatment-resistant schizophrenia? A factor analytic investigation based on data from the Pattern cohort study." Psychiatry Res 276: 210-217.	Pyramidical 4-factor model by Kay and Sevy, 1990	4	0.944	/	(-)
	Pentagonal 5-factor model by White et el. 1997	5	0.966	/	**(+)**
	van der Gaag et al. (2006b) model	5	0.966	/	**(+)**
	Wallwork et al. (2012) model (NIMH)	5	0.959	/	**(+)**
	Lindenmayer et el., 2004 model	5	0.968	/	**(+)**
	Present model	5	0.967	/	**(+)**
	Pyramidical 4-factor model by Kay and Sevy, 1990	4	0.954	/	**(+)**
	Pentagonal 5-factor model by White et el. 1997	5	0.969	/	**(+)**
	van der Gaag et al. (2006b) model	5	0.969	/	**(+)**
	Wallwork et al. (2012) model (NIMH)	5	0.964	/	**(+)**
	Lindenmayer et el., 2004 model	5	0.970	/	**(+)**
	Present model	5	0.972	/	**(+)**
	Pyramidical 4-factor model by Kay and Sevy, 1990	4	0.912	/	(-)
	Pentagonal 5-factor model by White et el. 1997	5	0.954	/	**(+)**
	van der Gaag et al. (2006b) model	5	0.955	/	**(+)**

CFAs of other models than Marder or the Three-factor Kay model					
reference	model	Factors	CFI	RMSEA	*Updated criteria* rating[a]
	Wallwork et al. (2012) model (NIMH)	5	0.945	/	(-)
	Lindenmayer et el., 2004 model	5	0.958	/	(+)
	Present model	5	0.946	/	(-)
Higuchi, C. H., et al. (2022). "Identifying strategies to improve PANSS based dimensional models in schizophrenia: Accounting for multilevel structure, Bayesian model and clinical staging." Schizophr Res 243: 424-430.	Wallwork 5-factor model	5	0.921	/	(-)
	Wallwork 5-factor model	5	0.975	/	(+)
	Wallwork 5-factor model	5	0.952	/	(+)
Nakaya, M., et al. (1999). "Latent structures underlying schizophrenic symptoms: a five-dimensional model." Schizophr Res 39(1): 39-50.	One-dimensional	1	NFIs: 0.64	/	(-)
	Two-dimensional Kay et al. (1987)	2	0.75	/	(-)
	Three-dimensional Strauss et al. (1974)	3	0.93	/	(-)
	Four-dimensional Kay and Sevy (1990)	4	0.74	/	(-)
	Four-dimensional Peralta and Cuesta (1994)	4	0.87	/	(-)
	(1994)	5	0.78	/	(-)
	Five-dimensional Modified Peralta and Cuesta (1994) model	5	0.91	/	(-)
	One-dimensional	1	0.62	/	(-)
	Two-dimensional Kay et al. (1987)	2	0.73	/	(-)
	Three-dimensional Strauss et al. (1974)	3	0.87	/	(-)
	Four-dimensional Kay and Sevy (1990)	4	0.81	/	(-)
	Four-dimensional Peralta and Cuesta (1994)	4	0.88	/	(-)
	Five-dimensional Lindenmayer et al. (1994)	5	0.77	/	(-)
	Five-dimensional Modified Peralta and Cuesta (1994) model	5	0.91	/	(-)
Lykouras, L., et al. (2000). "Five-factor model of schizophrenic psychopathology: how valid is it?" Eur Arch Psychiatry Clin Neurosci 250(2): 93-100.	Lindenmayer et al. (1995a) model, 5 factors	5	0.650	/	(-)
	Model derived from the present study, 7 factors	7	0.575	/	(-)
	Refined model derived from the present study, 5 factors	5	0.611	/	(-)
Drake, R. J., et al. (2003). "The evolution of symptoms in the early course of non-affective psychosis." Schizophr Res 63(1-2): 171-179.	Baseline PCA	5	NFIs: 0.93	/	(-)
	Final PCA (5 factors)	5	0.88	/	(-)
	Final PCA (6 factors)	6	0.88	/	(-)
	Change PCA	5	0.95	/	(-)
	Kay and Sevy, 1990	4	0.86	/	(-)
	Lindenmayer et al., 1994	5	0.11	/	(-)
	White et al., 1997	5	0.87	/	(-)
	Lancon et al., 1998 (chronic)	5	0.80	/	(-)
	Lancon et al. 2000 (acute)	5	0.75	/	(-)
	Mass et al., 2000	5	0.71	/	(-)
	Nakaya et al., 1999	5	0.84	/	(-)
Reichenberg, A., et al. (2005). "Stability in schizophrenia symptoms over time: findings from the Mount Sinai Pilgrim Psychiatric Center Longitudinal Study." J Abnorm Psychol 114(3): 363-	EFA derived 6-factor model time point 1	6	0.96	/	(+)
	EFA derived 6-factor model time point 2	6	0.95	/	(-)
Stochl, J., et al. (2014). "Multilevel ordinal factor analysis of the positive and negative syndrome scale (PANSS)." Int J Methods Psychiatr Res 23(1): 25-35.	Van den Oord et al. (2006)	5	0.609	0.145	(-)
	Bell et al. (1994a)	5	0.480	0.161	(-)
	Bell et al. (1994b)	5	0.464	0.169	(-)
	Dollfus and Petit (1995)	5	0.328	0.214	(-)
	Dudek (2005)	5	0.550	0.163	(-)
	Emsley et al. (2003)	5	0.851	0.088	(-)

CFAs of other models than Marder or the Three-factor Kay model					
reference	model	Factors	CFI	RMSEA	*Updated criteria* rating[a]
	White et al. (1997)	5	0.896	0.080	(-)
	Fredrikson et al. (1997)	5	0.542	0.166	(-)
	Higashima et al. (1998)	5	0.556	0.175	(-)
	Lancon et al. (1998)	5	0.850	0.100	(-)
	Lancon et al. (1999)	5	0.530	0.154	(-)
	Lancon et al. (2000)	5	0.643	0.154	(-)
	Lee et al. (2003)	5	0.560	0.153	(-)
	Levine and Rabinowitz (2007)	5	0.844	0.089	(-)
	Lykouras et al. (2000)	5	0.608	0.157	(-)
	Kay and Sevy (1990)	4	0.515	0.158	(-)
	Mass et al. (2000)	5	0.841	0.107	(-)
	Van den Oord et al. (2006)	5	0.630	0.140	(-)
	Wolthaus et al. (2000)	5	0.846	0.091	(-)
	Fredrikson et al. (1997)	5	0.456	0.179	(-)
	Van den Oord et al. (2006)	5	0.595	0.145	(-)
	Reininghaus et al. (2013)	6	0.868	0.085	(-)
	Emsley et al. (2003)	5	0.817	0.098	(-)
	Peralta and Cuesta (1994)	8	0.828	0.100	(-)
Anderson, A. E., et al. (2017). "Disparity between General Symptom Relief and Remission Criteria in the Positive and Negative Syndrome Scale (PANSS): A Post-treatment Bifactor Item Response Theory Model." Innov Clin Neurosci 14(11-12): 41-53.	unidimensional model: 5 factor model two factors like Marder: negative fact	5	0.917	/	(-)
	Bifactor model: G Factor with all items and 5 additional Factors	Bifactorial[b]	0.97	/	**(+)**
Findling, R. L., et al. (2023). "An Optimized Version of the Positive and Negative Symptoms Scale (PANSS) for Pediatric Trials." J Am Acad Child Adolesc Psychiatry 62(4): 427-434.	5-factor model, not further specified	5	0.73	/	(-)
Cuesta, M. J. and V. Peralta (1995). "Psychopathological dimensions in schizophrenia." Schizophrenia bulletin 21(3): 473-482.	One-dimensional Model	1	NFI: 0.657	/	(-)
	Two-dimensional (Kay et al. 1986b, 1987)	2	0.724	/	(-)
	Three-dimensional Peralta et al. (1992)	3	0.917	/	(-)
	Four-dimensional Pyramidical (Kay 1990, Kay et al. 1990)	4	0.876	/	(-)
	Four-dimensional Peralta and Cuesta (1994)	4	0.903	/	(-)
	Four-dimensional Strauss-Peralta (Strauss et al. 1974; Peralta et al. 1992)	4	0.927	/	(-)
Dollfus, S. and B. Everitt (1998). "Symptom structure in schizophrenia: two-, three- or four-factor models?" Psychopathology 31(3): 120-130.	Two-dimensional model derived from Lindenmayer et al. 1995	2	0.798	/	(-)
	Three-dimensional model derived from Lindenmayer et al. 1995	3	0.828	/	(-)
	Five-dimensional model derived from Lindenmayer et al. 1995	5	0.688	/	(-)

All models carry the names used by the respective studies authors. If not otherwise specified CFIs are in the CFI column, studies using NFIs instead are indicated by a 'NFI:' in the first row of the respective study. fit indices highlighted light grey surpass the CFI≥0.90 criterion, fit indices highlited dark grey surpass COSMINs CFI>0.95 criterion. [a]Results are rated according to the COSMIN updated criteria of good measurement properties ((+)-rating if CFI/NFI>0.95 or RMSEA<0.06). (+)-ratings are bold for better comprehensibility.[b]Bifactorial models contain 1 general factor all items are a priori assigned to and 5 specific factors between which the items are being distributed.
